# Decoding the heterogeneity of Alzheimer’s disease diagnosis and progression using multilayer networks

**DOI:** 10.1038/s41380-022-01886-z

**Published:** 2022-12-20

**Authors:** Bárbara Avelar-Pereira, Michael E. Belloy, Ruth O’Hara, S. M. Hadi Hosseini, for the Alzheimer’s Disease Neuroimaging Initiative

**Affiliations:** 1 Department of Psychiatry and Behavioral Sciences, School of Medicine, Stanford University, Stanford, CA 94304 USA; 2 Department of Neurology and Neurological Sciences, School of Medicine, Stanford University, Stanford, CA 94304 USA

**Keywords:** Neuroscience, Diseases

## Abstract

Alzheimer’s disease (AD) is a multifactorial and heterogeneous disorder, which makes early detection a challenge. Studies have attempted to combine biomarkers to improve AD detection and predict progression. However, most of the existing work reports results in parallel or compares normalized findings but does not analyze data simultaneously. We tested a multi-dimensional network framework, applied to 490 subjects (cognitively normal [CN] = 147; mild cognitive impairment [MCI] = 287; AD = 56) from ADNI, to create a single model capable of capturing the heterogeneity and progression of AD. First, we constructed subject similarity networks for structural magnetic resonance imaging, amyloid-β positron emission tomography, cerebrospinal fluid, cognition, and genetics data and then applied multilayer community detection to find groups with shared similarities across modalities. Individuals were also followed-up longitudinally, with AD subjects having, on average, 4.5 years of follow-up. Our findings show that multilayer community detection allows for accurate identification of present and future AD (≈90%) and is also able to identify cases that were misdiagnosed clinically. From all MCI participants who developed AD or reverted to CN, the multilayer model correctly identified 90.8% and 88.5% of cases respectively. We observed similar subtypes across the full sample and when examining multimodal data from subjects with no AD pathology (i.e., amyloid negative). Finally, these results were also validated using an independent testing set. In summary, the multilayer framework is successful in detecting AD and provides unique insight into the heterogeneity of the disease by identifying subtypes that share similar multidisciplinary profiles of neurological, cognitive, pathological, and genetics information.

## Introduction

Dementia is characterized by a progressive deterioration of all cognitive domains, with Alzheimer’s disease (AD) accounting for the majority of cases [1–3]. Detecting at-risk individuals early can allow for more effective treatment and therapy and delay the onset and slow down disease progression. Hence, past research has largely focused on establishing AD-related biomarkers during preclinical stages [4–6]. Several biomarkers have been identified using neuroimaging, genetics, and behavioral data. These include risk factors such as inheritance of the ε4 allele of the *apolipoprotein E* (APOE ε4) gene [7, 8], age [2, 9], family history [10, 11], and lifestyle [12–14], but also imaging biomarkers based on positron emission tomography (PET) and magnetic resonance imaging (MRI) [15, 16]. Amyloid and tau burden, which correspond to the accumulation of amyloid-β (Aβ) plaques and tau neurofibrillary tangles (NFTs) respectively, can be detected through PET and in cerebrospinal fluid (CSF) [17, 18]. Brain atrophy measured with structural MRI is also a well-established marker of AD and correlates with disease severity [19, 20]. In addition – and given that cognitive problems are a validated characteristic of the disease – cognitive performance measurements, especially of episodic memory but also perceptual speed and executive functioning, are commonly used in diagnosis [21–23].

These advances have shifted our understanding of AD from a purely clinical and symptom-based disease to a biological construct that is both multifactorial and heterogenous. The above-mentioned modalities have, individually or in conjunction with others, been shown to predict AD to different extents and at different stages of development, which suggests that each modality conveys unique variance but also indicates the need for a multifactorial approach [1, 3, 5, 19, 24–27]. In line with this are postmortem findings of elderly individuals with high amyloid burden and NFTs deposition but without clinical AD signs [28–31]. These, together with studies that utilized different machine learning algorithms [32–34], confirm that AD cannot be explained by any single biomarker. Furthermore, despite the wealth of available data, it remains challenging to find an adequate balance between data availability, model complexity, and interpretability. There are numerous studies combining data from different biomarkers, but a limitation of such approaches is that they typically report results in parallel or compare normalized findings but do not analyze them *simultaneously* [35].

Here, we aimed to test a multi-dimensional network framework which aggregates data across a range of modalities into a single model capable of capturing the heterogeneity of AD. The superiority of this technique over standard approaches has been demonstrated in other fields, namely cancer, where it has been able to identify cancer subtypes and predict survival [36]. We applied this framework to 490 subjects from the Alzheimer’s Disease Neuroimaging Initiative (ADNI) who had data across five modalities including MRI, PET, CSF, genetics, and cognition at baseline. These modalities were chosen with the goal of covering well-established AD markers while ensuring a reasonable sample size. Overall, we hypothesized that, by detecting common as well as complementary signals across modalities and minimizing the effect of different scales and noise, the multilayer network framework would provide unique insight regarding AD development and progression. We also validated our findings by dividing the sample chronologically into a training and testing set to ensure results were reliable.

The current consensus on AD dementia is that individuals are first in a preclinical stage (e.g., asymptomatic) and later move on to mild cognitive impairment (MCI) and finally AD [27]. Individuals with MCI were categorized into early (EMCI) or late (LMCI) MCI, depending on how poorly they performed on cognitive screening tools [37]. LMCI has the highest risk of developing AD, with EMCI displaying the lowest conversion rate [37–39]. The need for a definition of early and late MCI relates to the fact that cognitive deficits in AD are initially subtle and, thus, not detected via traditional cognitive assessments but become more evident the closer one is to diagnosis. This classification allows for a logical progression from cognitively normal (CN) to preclinical, prodromal, and finally AD dementia [38, 40]. We expect to be able to accurately detect present and future CN, MCI, and AD cases across the sample, but also provide novel information into the biological underpinnings that lead to AD. As such, we also divided subjects into amyloid positive and negative groups using baseline amyloid-β PET and analyzed them separately. This was done under the assumption that amyloid positive individuals already display severe AD-related pathology as measured by PET. Given that one of our primary goals is detecting the disease early on, these subsamples allow us to test the model at different stages of disease severity.

## Materials and methods

### Participants

Data used in preparation of this work were obtained from the ADNI database (http://adni.loni.usc.edu). ADNI is a multicenter study launched in 2003, designed with the goal of developing and validating biomarkers for early detection and treatment of AD. Details on overall inclusion and exclusion criteria can be found elsewhere [41]. Informed consent was obtained from all participants or their authorized representatives.

The study was based on 490 subjects (age 71.80 ± 7.07, 55–90.3, 231 women) who had data across five modalities at baseline and which are described in the following sections. The sample was comprised of CN, MCI, and AD individuals (for details on participants’ characteristics at baseline see Table 1). An MCI diagnosis was based on the Petersen criteria such that individuals showed a Mini-Mental State Examination (MMSE) [42] score between 24–30, subjective memory concerns, abnormal memory function measured by the Logical Memory II subscale of the Wechsler Memory Scale [43], a clinical dementia rating of 0.5 [44], absence of significant impairment in other cognitive domains, and absence of dementia. The subdivision between EMCI and LMCI was done based on an episodic memory test. Participants were considered EMCI or LMCI if their memory scores were between 1–1.5 SD or at least 1.5 SD below their education-adjusted means, respectively. Nomenclature has changed over the years, but LMCI can be considered amnestic MCI. Participants were considered AD if they met the NINCDS-ADRDA criteria for probable AD [45, 46].

**Table 1:** Baseline characteristics by group.

	CN	MCI	AD	Group differences
*n*	147	287	56	
Mean age ± SD	73.12 ± 5.92	70.63 ± 7.12	74.11 ± 8.33	CN vs. MCI: *p* < 0.001 CN vs. AD: *p* = 0.445 MCI vs. AD: *p* = 0.001
Sex (female/male)	78/69	130/157	23/33	CN vs. MCI: *p* = 0.193 CN vs. AD: *p* = 0.127 MCI vs. AD: *p* = 0.561
Years of education ± SD	16.56 ± 2.52	16.37 ± 262	15.64 ± 2.68	CN vs. MCI: *p* = 0.472 CN vs. AD: *p* = 0.024 MCI vs. AD: *p* = 0.059
Mean MMSE score ± SD	29.07 ± 1.11	28.16 ± 1.69	23.32 ± 1.85	CN vs. MCI: *p* < 0.001 CN vs. AD: *p* < 0.001 MCI vs. AD: *p* < 0.001

### Modalities included in the multilayer network framework

#### Structural MRI

Structural images were acquired using a standardized 3 T protocol in a GE, Siemens, or Phillips scanner with a 32-channel coil. Cortical reconstruction and volumetric segmentation were performed with FreeSurfer (version 5.1) by the University of California, San Francisco (UCSF) Medical Center team [47, 48] and are available on the ADNI website. For further details regarding MRI acquisition, preprocessing pipeline, and quality control procedures, see the UCSF FreeSurfer Methods (https://ida.loni.usc.edu). For a summary table of the regions of interest [ROIs] used see S1.

#### Amyloid-β PET

Baseline Aβ data were obtained using ^18^F-AV-45 (florbetapir) PET. Details on the acquisition protocol and preprocessing are available on the ADNI website (http://adni.loni.usc.edu/methods/pet-analysis-method/pet-analysis/). In summary, florbetapir scans were co-registered to each subject structural MRI scan and standardized uptake value ratios (SUVR) were extracted from each ROI. A reference region (i.e., whole cerebellum) was used for normalization [49, 50]. We also divided the sample into amyloid positive/negative individuals by applying a cutoff of 1.11 [51] (see S1 for ROIS used).

#### CSF

This modality was comprised of amyloid-β (Aβ_1-42_), total Tau (Tau), and phosphorylated p-TAU_181p_ (pTau). All samples, taken from lumbar punctures performed as described in the ADNI manual, were shipped to the ADNI Biomarker Core at the University of Pennsylvania School of Medicine [52, 53]. CSF concentrations were measured using the multiplex xMAP Luminex platform (Luminex Corp., TX, USA) with the INNO-BIA AlzBio3 kit (Innogenetics, Belgium). Details on the standardized protocol for CSF analysis are also available elsewhere (http://www.adni-info.org).

#### Cognition

Cognitive data included the Rey Auditory Verbal Learning Test [54], Trail Making Test [55], Everyday Cognition and Study Partner [56], and Functional Assessment Questionnaire [57]. These reflect domain-specific measures of memory, executive functioning, self- and study partner reports of cognitive function, and daily independence. We purposefully refrained from including scores of global cognition such as the MMSE, Montreal Cognitive Assessment (MoCA) [58], or Clinical Dementia Rating Scale Sum of Boxes (CDRSB) as these are typically used for diagnostic purposes (see Participants) and would, therefore, lead to high specificity but be biased and of circular nature when included in the multilayer network. As a form of validation and to interpret the multilayer communities, we report group differences for these cognitive scores in the results.

#### Genetics

This layer included participants APOE information and their polygenic hazard score (PHS). This score is derived from 31 single nucleotide polymorphisms (SNPs) and has been shown to reliably identify those who are at risk for AD at any age [59]. It also provides additional information beyond the risk associated with the APOE ε4 allele.

### Multilayer network construction

Before carrying out network construction, all data underwent three preprocessing steps: mean centering, regressing out covariates, and normalization. This was done separately for each modality. For structural MRI and amyloid-β PET, age, gender, and total intracranial volume (ICV) were used as covariates, whereas for CSF and cognition only age and gender were considered and, for genetics, only gender was used. Max normalization (data divided by max value across subjects) was carried out to scale data between 0 and 1, except for genetics in which *z*-scores were used instead. The normalized data for each modality were used to create a subject similarity network with nodes representing individuals and edges representing the similarity of individuals across features quantified using pair-wise correlations.

Next, a multi-dimensional network framework (i.e., a multilayer network) was constructed based on the work by Mucha et al., 2010 [60], where the authors generalized the concept of community detection for multiplex networks. This algorithm has been shown to detect communities that are robust relative to null models. Each node represents the manifestation of a given entity (i.e., subject) at a given layer (i.e., modality) with edges representing connections within and between layers. All individuals were present at each layer, but their interactions were allowed to vary across modalities. Several metrics can be derived to describe such networks but, given that one of the study goals was to explore AD heterogeneity, modularity is of particular importance since it captures strength of partition in the network. Modularity was derived using an iterative generalized Louvain (GenLouvain) community detection algorithm which is optimized for the study of multilayer/multiplex networks. The modularity quality function (*Q*) is defined as:

$$Q = \frac{1}{{2\mu }}\mathop {\sum}\limits_{ijlr} {\left\{ {\left( {A_{ijl} - \gamma _lM_{ijl}} \right)\delta _{lr} + \delta _{ij}\omega _{jlr}} \right\}} \left( {\delta \left( {g_{il},g_{jr}} \right)} \right)$$

where the matrix of layer *l* has components $A_{ijl}$ and $M_{ijl}$ gives the corresponding components for the optimization null model. The structural resolution or scaling parameter ($\gamma$) of layer *l* is $\gamma _l$, $g_{il}$ and $g_{jr}$ are the community assignments of node *i* in layer *l* and node *j* in layer *r*, $\omega _{jlr}$ corresponds to the interlayer coupling strength parameter connecting node *j* in layers *l* and *r*. Finally, *μ* is the total edge weight in the network. For additional details, see S2.

### Statistical analyses

We used analysis of variance (ANOVAs), independent-sample *t*-tests, chi-square tests and Whitney–Mann *U* tests to compute differences between communities in the variables of interest. These included age, education, and APOE status, but also cognitive measurements such as the MMSE, MoCA, CDRSB, Alzheimer’s Disease Assessment Scale (ADAS-Cog13) [61, 62], and ADNI’s scores using Item Response Theory for memory and executive functioning [63, 64]. In addition, we compared subjects’ brain volume in the hippocampus, entorhinal cortex, and whole brain as well as amyloid load using PET and tau, pTau, and amyloid using CSF. When appropriate, ANOVAs were followed by post-hoc *t*-tests corrected for multiple comparisons using Bonferroni correction (*p*_*corrected*_ < 0.05). A validation analysis was carried out by splitting the sample and building a model using the older data (collected between 06/2010 and 04/2012) and tested on more newly acquired data (collected between 04/2012 and 10/2013).

## Results

### Multilayer network identifies healthy and Alzheimer’s disease cases accurately

**Fig. 1:**
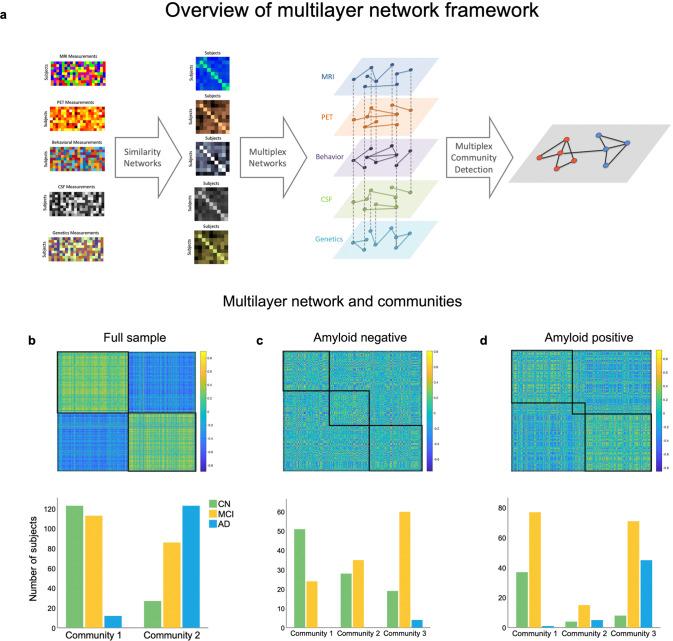
Overview of the multilayer network framework used in the study and resulting communities. **a** The first column displays each individual modality included in the multilayer network model (subjects x features), while the second column shows the corresponding similarity networks (subjects x subjects). In the third column, the multilayer network is displayed, with each diamond representing a layer, solid lines representing intralayer interactions, and dotted lines representing interlayer interactions. Nodes are connected across and within layers. The last column exemplifies multilayer community detection, where two communities are identified based on communalities in the data. **b** Multilayer network communities and distribution for each diagnosis group across the entire sample, (**c**) in the amyloid negative subsample, and (**d**) in the amyloid positive subsample. The matrices represent the similarity between subjects across features quantified using pair-wise correlations.

The multilayer network divided the sample into two communities (Fig. 1a, b; see Table 2 for an overview of subjects’ baseline characteristics), one including 91.1% of all AD cases and the other the majority (82%) of CN individuals (S3 for monolayer results). Sensitivity and specificity for CN and AD individuals were 92.98% and 76.19% (95% CI: 83–98.05 and 68.47–82.82) at baseline and 91.1% and 82% (95% CI 84.99–95.32 and 74.9–87.79) for final diagnosis, respectively. We investigated how the communities were distinct from each other, with comparisons being regarded as significant if they survived Bonferroni correction (*p* = 0.00357). The communities differed in a multitude of relevant characteristics for AD, including APOE status (χ(4) = 127.885, *p* = 1.10 × 10^−26^), with those in community 1 having less participants with one or two ε4 allele(s). However, they were not different in age (*t*(488) = −2.542, *p* = 0.011) or education (*t*(488) = 2.230, *p* = 0.026). We also compared them on a range of clinical and cognitive assessments, with community 2 showing worse performance in the MMSE (*t*(368.102) = 10.250, *p* = 7.46 × 10^−22^), MoCA (*t*(435.405) = 8.759, *p* = 4.423 × 10^−17^), CDRSB (χ(15) = 115.65, *p* = 1.31 × 10^−17^), and ADAS13 (*t*(395.127) = −11.562, *p* = 7.83 × 10^−27^). The same pattern was observed in memory (*t*(460.267) = 13.339, *p* = 1.5 × 10^−35^) and executive functioning (*t*(480) = 9.314, *p* = 1.86 × 10^−18^). In addition, the groups differed in brain imaging measurements with community 2 showing more signs of atrophy in the hippocampus (*t*(434.516) = 8.924, *p* = 1.27 × 10^−17^), entorhinal cortex (*t*(465.730) = 7.159, *p* = 3.18 × 10^−12^) and in the whole brain (*t* = 3.09, *p* = 0.002). Those in community 2 also had higher CSF concentrations of tau (*t*(305.314) = −14.568, *p* = 7.3 × 10^−37^) and pTau (*t*(378.678) = −15.099, *p* = 1.18 × 10^−40^), and lower amyloid-β (*t*(408.280) = 25.382, *p* = 5.55 × 10^−86^). Finally, community 2 had a higher Aβ burden (*t*(323.571) = −30.122, *p* = 0.002) measured by PET.

**Table 2:** Summary of results.

Multilayer communities				
		1	2	Total
Diagnosis	CN % within diagnosis	123 82%	27 18%	150 100%
	MCI % within diagnosis	113 56.8%	86 43.2%	199 100%
	AD % within diagnosis	12 8.9%	123 91.1%	135 100%

Although the distribution of MCI was more skewed towards including these subjects in community 1, they were still rather split (56.8% vs. 42.3%). As such, we examined MCI subtypes and found that 69.5% of LMCI, which have the highest probability of progressing to AD, were part of community 2. The opposite was true for EMCI, with only 30.5% of individuals belonging to this same community. Out of the 26 participants who, at their final available follow-up had reverted to CN, 88.5% were part of community 1, suggesting that those with more severe pathology were correctly identified as part of the second community. Likewise, individuals who were possibly misdiagnosed or had an MCI unrelated to AD had a higher likelihood of being categorized in community 1.

**Fig. 2:**
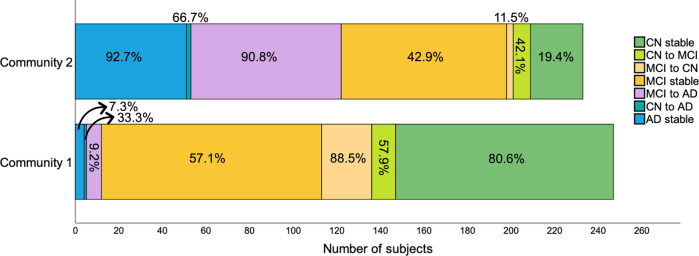
Community 1 and 2 divided by change in diagnosis across the full sample. Percentages correspond to the proportion of individuals within each category that belong to community 1 or 2.

We then examined how communities differed in progression to AD (Fig. 2). Overall, the average follow-up period for CN individuals was 6 ± 2.7 years and for MCI/AD individuals was 4.5 ± 2.5 years. From all MCI participants who developed AD, 9.2% belonged to community 1 and 90.8% belonged to community 2, indicating that the multilayer method was not only able to detect present but also future AD. Community 2 included most subjects who remained with AD throughout the study but also those who progressed to AD from a CN or an MCI status. Of note, even though community 1 included more stable MCI individuals (57.1%), many of these were EMCI (61.7% in community 1). This was expected since subjects who were both MCI at baseline and part of community 2 eventually progressed to AD (42.7%). Still, for a comparison of stable MCI between community 1 and 2, see S4.

**Fig. 3:**
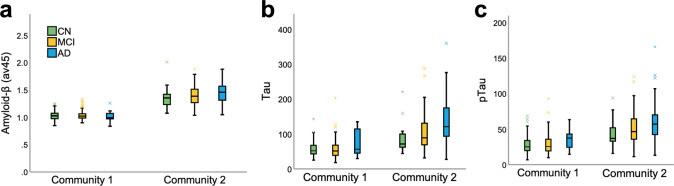
Amyloid and tau load. **a** PET amyloid load (av45), (**b**) CSF tau, and (**c**) CSF pTau for community 1 and 2 by diagnosis group.

Next, we focused on outlier subjects, i.e., those whose diagnosis did not match their community allocation. AD subjects who were “misclassified” and placed in community 1 (*n* = 12) had a distinctive signature compared to those in community 2 (i.e., AD dominant; *n* = 123). These subjects correspond to a small group of individuals and, as such, we report descriptive statistics and nonparametric tests (see S5) when comparing them to the rest of the AD sample. In summary, these 12 AD individuals displayed lower CSF tau and pTau, higher CSF amyloid-β, and lower amyloid accumulation in the brain when compared to AD cases in community 2 (Fig. 3). Even when compared to CN (M = 1.03, SD = 0.01) and MCI (M = 1.04, SD = 0.08) individuals within their own community, this group displayed lower amyloid-β concentrations. This suggests that the multilayer methodology is sensitive in identifying individuals who, although diagnosed as AD based on neuropsychological assessments, did not meet other AD criteria, and was able to label them as part of the CN-dominant community.

We also investigated mismatched subjects who were part of community 2 but were deemed as CN. There were 27 subjects who were CN (18%) in community 2 and 123 (82%) in community 1. Given the difference in sample size, we compared the groups using nonparametric statistics and reported descriptive values (see S5). Interestingly, healthy subjects in community 2 had higher CSF tau and pTau, lower CSF amyloid-β, and higher amyloid-β accumulation in the brain when compared to their CN counterparts in community 1 (Fig. 3). Importantly, the validation analysis also confirmed the results obtained across the full sample (see S6). Likewise, we followed up our sample yearly from 12 to 48 months and at final diagnosis and found that the model was successful in distinguishing MCI conversion to AD and reversion to CN over time. For converters and reverters, specificity, sensitivity, and accuracy were all above 85% at final diagnosis (S7).

**Fig. 4:**
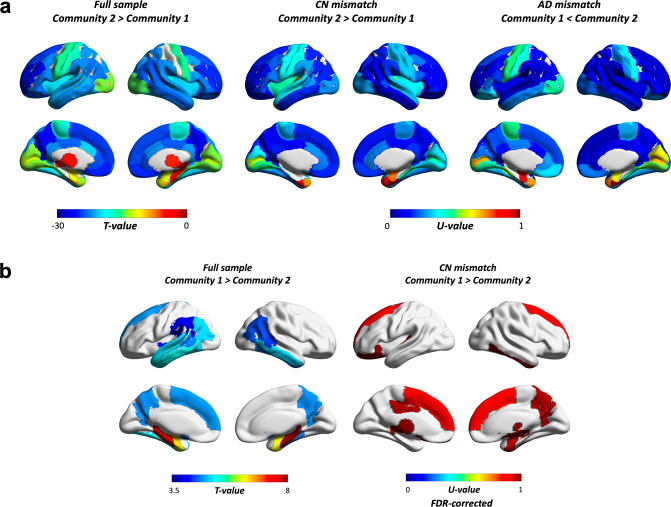
Differences across the brain in. **a** PET amyloid load and (**b**) volume. The full sample compares all subjects in community 1 (i.e., CN dominant) to all subjects in community 2 (i.e., AD dominant), whereas CN and AD mismatches compare only CN (*N* = 123 vs. 27) or AD (*N* = 12 vs. 123) cases between communities. *U*-values were scaled (divided by maximum *U*) for ease of interpretation. Results were Bonferroni corrected unless otherwise stated (all except for volumetric findings in CN mismatched cases, which were FDR corrected instead). Masks were weighted using results of *t*-tests or Whitney–Mann *U* tests. These were based on ROIs from the Desikan-Killiany Atlas, and included the amygdala, nucleus accumbens, hippocampus, pallidum, thalamus, caudate, and ventral diencephalon (ventral DC).

We further examined whether communities showed differential spatial patterns in amyloid and volumetric estimates. Results showed that the AD-dominant community had a higher amyloid burden across most of the brain compared to the CN-dominant community (Fig. 4a). This distributed pattern was also present in CN and AD mismatched findings (Fig. 4a, b for amyloid and structural MRI measures respectively). Among regions showing the largest differences in the full sample were the precuneus, parts of orbitofrontal, superior frontal, rostral anterior cingulate and middle frontal, left posterior cingulate, left frontal pole, right inferior parietal, and right middle temporal cortex. A similar scenario was seen in CN-mismatched individuals, where CN subjects allocated to the AD-dominant community showed the highest amyloid differences when compared to CN in the “healthier” community in many of the same regions. These included the precuneus, parts of the orbitofrontal, rostral middle frontal, posterior cingulate, right inferior parietal and middle temporal cortex. Several of these areas are established as parts of the brain where amyloid first starts to accumulate. When comparing AD mismatched subjects, the largest differences included some overlap with the full sample (e.g., rostral middle frontal, right superior frontal, right posterior cingulate) but several of the regions were not the same, including the temporal sulcus, caudal middle frontal and supramarginal gyrus, superior temporal cortex, and insula.

We also found that across the sample, the AD-dominant community showed lower volume in the precuneus, basal ganglia (e.g., nucleus acccumbens, left putamen), medial temporal lobe (e.g., amygdala, hippocampus, parahippocampus, entorhinal cortex), temporal sulcus, fusiform, inferior and middle temporal, inferior parietal, left superior temporal and left frontal, and left supramarginal gyrus. CN individuals in the AD-dominant community also showed lower volume in partially the same regions (Fig. 4b “CN mismatch”), such as the right precuneus, basal ganglia (e.g., right pallidum, nucleus accumbens), medial temporal lobe (e.g., right amygdala, right entorhinal cortex), inferior temporal and left superior frontal gyrus. Other regions included the right superior frontal gyrus, ventral DC, lateral orbitofrontal and posterior cingulate cortex, left thalamus and transverse temporal gyrus. No differences survived correction for multiple comparisons in the AD-mismatched subsample.

### Multilayer network in amyloid negative subjects identifies different stages of cognitive impairment and brain pathology

Applying the multilayer method to the amyloid negative sample (*n* = 226) resulted in three communities (Fig. 1c). Community 1 had the largest number of CN subjects (52%), followed by community 2 (28.6%) and 3 (19.4%). The opposite was true for MCI, with community 1 having the smallest number of individuals (19.4%), followed by community 2 (29.8%). Thus, slightly more than half of all MCI cases (50.8%) were categorized as part of community 3. Both the number of EMCI and LMCI increased from community 1 (EMCI: 24.7%; LMCI: 3.2%) to 2 (EMCI: 29%; LMCI: 32% 3) and 3 (EMCI: 46.2%; LMCI: 64.5%) in a stepwise fashion. Given that these are individuals with a low amyloid burden, the number of AD cases was small but were all part of community 3 (*n* = 4; 100%).

We performed ANOVAs to test between-group differences, with comparisons being significant if they survived Bonferroni correction (*p* = 0.00313), followed by post-hoc *t*-tests when applicable. For most variables, there was a clear step-by-step pattern of increased pathology and lower behavioral performance from community 1 to community 3 (for a distribution of these variables see S8). Age (*F*(2,223) = 0.118, *p* = 0.889), education (*F*(2,223) = 0.709, *p* = 0.493), and APOE (χ(8) = 6.207, *p* = 0.624) were not different between groups, but MMSE (*F*(2,223) = 8.434, *p* = 0.0003), MoCA (*F*(2,220) = 21.424, *p* = 3.15 × 10^−9^), memory (*F*(2,221) = 47.916, *p* = 5.17 × 10^−18^), executive functioning (*F*(2,221) = 30.317, *p* = 2.32 × 10^−12^), ADAS13 (*F*(2,223) = 29.127, *p* = 5.77 × 10^−12^), and CDRSB (*F*(2,223) = 12.3, *p* = 9 × 10^−6^) were. Likewise, volume in the hippocampus (*F*(2,203) = 7.36, *p* = 0.001) was significantly different between communities, but entorhinal (*F*(2,223) = 4.115, *p* = 0.018) and whole brain (*F*(2,223) = 0.937, *p* = 0.393) volumes were not. Finally, CSF tau (*F*(2,221) = 13.172, *p* = 4 × 10^−6^) and amyloid PET (*F*(2,223) = 5.845, *p* = 0.003) showed significant differences but this was not the case for pTau (*F*(2,223) = 0.586, *p* = 0.558) and CSF amyloid (*F*(2,223) = 2.911, *p* = 0.056). In summary, for MMSE, scores in community 1 were not different than those in community 2, but subjects in community 3 had lower scores compared to 1 and 2. For memory, executive functioning, MoCA, and ADAS13, all communities were different from each other, with community 1 showing the best performance and 3 showing the worst. In CDRSB, there was an incremental increase, with community 1 and 2 showing similar scores, and 3 scoring the worst. Hippocampal volume was lower in community 3 compared to 1 and 2, but community 1 and 2 were identical. Of note, for both tau and amyloid PET, a different pattern emerged with community 2 showing the lowest mean values while community 1 and 3 were identical. For details on p-values for each comparison, see Table 3.

**Table 3:** Post-hoc t-tests among communities in amyloid negative subjects.

	Community 1	Community 2	Community 3	Group differences
Age	70.12 ± 6.2	70.63 ± 7.14	70.56 ± 7.43	1 vs. 2: *p* = 0.651 1 vs. 3: *p* = 0.683 2 vs. 3: *p* = 0.957
MMSE	29.20 ± 0.99	28.94 ± 1.40	28.30 ± 17.4	1 vs. 2: *p* = 0.211 1 vs. 3: *p* = 7.4 × 10^−5^ 2 vs. 3: *p* = 0.017
MoCA	26.36 ± 2.11	24.86 ± 283	23.49 ± 3.15	1 vs. 2: *p* = 0.001 1 vs. 3: *p* = 5.3 × 10^−10^ 2 vs. 3: *p* = 0.007
Memory	1.28 ± 0.55	0.88 ± 0.66	0.37 ± 0.56	1 vs. 2: *p* = 0.0002 1 vs. 3: *p* = 2.26 × 10^−19^ 2 vs. 3: *p* = 1 × 10^−6^
EF*	1.35 ± 0.75	0.822 ± 0.87	0.40 ± 0.72	1 vs. 2: *p* = 0.0002 1 vs. 3: *p* = 7.11 × 10^−14^ 2 vs. 3: *p* = 0.001
ADAS13	7.88 ± 3.55	11.08 ± 5.48	14.17 ± 6.16	1 vs. 2: *p* = 0.0001 1 vs. 3: *p* = 3.1 × 10^−13^ 2 vs. 3: *p* = 0.002
CDRSB	0.41 ± 0.74	0.70 ± 0.78	1.17 ± 1.25	1 vs. 2: *p* = 0.028 1 vs. 3: *p* = 6 × 10^−6^ 2 vs. 3: *p* = 0.005
Hippocampus	7714.55 ± 962.65	7608.20 ± 817.24	7102.93 ± 1212.92	1 vs. 2: *p* = 0.5 1 vs. 3: *p* = 0.001 2 vs. 3: *p* = 0.005
CSF Tau	62.69 ± 27.85	44.28 ± 14.83	62.22 ± 25.69	1 vs. 2: *p* = 2 × 10^−6^ 1 vs. 3: *p* = 0.912 2 vs. 3: *p* = 3.32 × 10^−7^
PET amyloid-β	1.02 ± 0.054	1.00 ± 0.48	10.2 ± 0.052	1 vs. 2: *p* = 0.015 1 vs. 3: *p* = 0.414 2 vs. 3: *p* = 0.001

*EF executive functioning.

Longitudinally, results were similar to the full sample. Community 1 corresponded to the healthiest group, with few CN subjects progressing to MCI. Community 2 seemed to reflect an intermediate stage with MCI individuals who remained stable, reverted to CN, or progressed to AD. Community 3 included all AD at baseline and the largest number of those who progressed to AD (S9).

### Multilayer network in amyloid positive subjects mimics findings on the full sample but with higher level of cognitive impairment and brain pathology

Our final analyses focused on the amyloid positive sample (Fig. 1d), where there was a similar trajectory to what was reported for the amyloid negative and full sample, albeit with a more pronounced pathology level given the higher amyloid burden (S10). Most healthy individuals were part of community 1 (75.5%) and most AD subjects (all but 1) were in community 3 (88.5%). Similarly, community 1 had the smallest number of AD cases (1.9%) and community 3 had only 16.3% of CN individuals. MCI was, again, relatively split between groups (47.2% vs. 43.6%) but the majority of participants with EMCI were in community 1 (62.9%), whereas the majority of LMCI were in community 2 (60.8%).

We found that, compared to those in community 3, individuals in community 1 had a better APOE profile (χ(4) = 28.659, *p* = 9 × 10^−6^), and higher behavioral performance measured by MMSE (*t*(184.792) = 10.410, *p* = 3 × 10^−20^), MoCA (*t*(213.612) = 9.718, *p* = 1.01 × 10^−18^), memory (*t*(232) = 13.863, *p* = 3.08 × 10^−32^), executive functioning (*t*(232) = 7.863, *p* = 1.41 × 10^−13^), ADAS13 (*t*(210.479) = −12.510, *p* = 2.27 × 10^−27^), and CDRSB (χ(15) = 76.952, *p* = 2.51 × 10^−10^). They also had higher hippocampal (*t*(220) = 9.66, *p* = 1.2 × 10^−18^) and entorhinal (*t*(232.577) = 6.827, *p* = 7.5 × 10^−11^) volumes, but whole brain volume (*t*(236) = 2.762, *p* = 0.006) did not survive Bonferroni correction. Additionally, they had lower CSF tau (*t*(198.599) = −8.611, *p* = 2.2 × 10^−15^) and pTau (*t*(237.683) = −6.516, *p* = 4.26 × 10^−10^) and, similar to what was reported for the full sample, those in community 1 had higher CSF amyloid (*t*(177.818) = 8.193, *p* = 4.84 × 10^−14^) and lower amyloid PET (*t*(238) = −8.269, *p* = 9.57 × 10^−15^). Age was not different between groups (*t*(231.832) = −0.465, *p* = 0.642). Community 3 included the majority of MCI cases that progressed to AD. Community 1 included most stable CN but also most individuals who progressed to MCI and, although all individuals were amyloid positive, had a small number of AD subjects (S9).

Of note, the results included three communities, not two. Both 1 and 3 are reported above. Community 2 is smaller (*n* = 24) and seemed to mimic the relative distribution of community 3 with few healthy subjects (*n* = 4), followed by a larger number of MCI (*n* = 15) and a comparatively large number of AD cases (*n* = 5). It seems to be a rather heterogenous group that, for most measures, falls between community 1 and 3.

## Discussion

Our results indicate that the multilayer technique is promising for both capturing the heterogeneity that exists across the AD spectrum and determining which subjects have or will develop AD. Moreover, we were able to predict conversion from MCI to CN. The advantages of such a network compared with traditional approaches is that it models each modality as a layer of a multi-dimensional network and, as such, is equipped to handle interactions *across* modalities and find relationships which might otherwise have been overlooked. Thus, similarly to what has been reported in other fields [36], we were able to predict with high accuracy and with only baseline data the individuals who remained cognitively healthy and those who did not.

The majority of CN and MCI cases who eventually progressed to AD were allocated to the AD-dominant community. In particular, the ability to successfully discriminate MCI converters (e.g., those who progressed to AD) and reverters (e.g., those who returned to a CN status) using only baseline multimodal data is noteworthy. This is a difficult task and literature shows that individuals who revert to CN can fluctuate and be, once again, classified as MCI at a follow-up examination. However, in our study, almost all MCI individuals who reverted to a CN status and remained as such were correctly identified as belonging to the CN-dominant community. This suggests that the multilayer model was able to identify cases of MCI unrelated to AD dementia and those displaying signs of “healthy aging” [37, 65–69]. Remarkably, we also followed up our sample yearly from 12 to 48 months and at final diagnosis and found that the model was successful in distinguishing MCI conversion to AD and reversion to CN over time. For converters and reverters, specificity, sensitivity, and accuracy were all above 85% at final diagnosis (S7).

By exploring the composition of the identified communities, we found that they differed in measurements relevant for diagnosis, including cognitive scores and brain biomarkers [2, 15, 16, 24]. Results were consistent across neuropsychological assessment tools, which is noteworthy given that common AD screening tests were not included in the model [70]. Our findings were also consistent across volumetric and Aβ and tau proxies [19, 25, 26, 71]. Even when dividing the sample into amyloid positive and negative, similar patterns identified for the full sample emerged, albeit with more or less pronounced levels of pathology. We can understand these findings by considering that the multilayer network labelled communities as representing individuals with low levels of pathology and good cognitive performance and who, therefore, maintained a “healthy aging” status, versus those who, comparatively, had higher levels of pathology and lower behavioral performance and were considered as showing more evidence of “AD-related aging” [72]. Importantly, the model was able to use this information to predict not only which individuals with cognitive impairment would develop AD, but also those who did not. We emphasize AD specifically because that is what the modalities are tailored to identify. If, instead, the goal had been to detect other types of dementia, the focus of each modality (e.g., cognitive assessments) or the modality itself (e.g., PET with a different radioligand) would have to change as well and be adjusted to detect those disorders instead. The fact that most LMCI cases were part of the AD-dominant community and that almost all of those who reverted to CN were part of the “healthier” community gives strength to the premise that the method is specifically detecting AD and not age-related neurodegeneration or cognitive decline in general [37, 73, 74]. It is also possible that the few mismatched cases of AD might be explained by other types of dementia that are sometimes confounded with AD itself [69, 75, 76].

In line with this premise is the fact that the communities did not entirely overlap with diagnosis from the NINCDS-ADRDA criteria. When examining “mismatched” CN controls and AD patients, it became apparent that they displayed a distinct signature when compared to others within their diagnosis group. AD cases in the CN-dominant community showed better outcomes than AD cases in the AD-dominant community for almost all comparisons, but also lower Aβ burden. This suggests that the multilayer framework identified subjects who did not meet AD criteria in regard to non-cognitive markers and labelled them as part of the healthier community [28, 30, 31]. Similarly, CN participants in the AD-dominant community showed worse outcomes in most variables of interest, including higher deposition of Aβ and tau in the brain [17, 77]. The spatial patterns identified by amyloid and structural MRI are consistent with this evidence. Amyloid burden across the brain was higher in the AD-dominant community, but also higher in CN individuals allocated to that same community when compared to their CN counterparts in community 1. Several of these areas are well-known parts of the brain where amyloid and even tau first start to accumulate such as the precuneus, inferior temporal gyrus, amygdala or entorhinal cortex [78–80]. Similar results can be found for volume, although to a lesser extent. Such findings suggest that the multilayer network recognized non-AD subjects who showed a similar signature to those who already have AD dementia and allocated them to the same community. Amyloid is known to accumulate decades before the presence of any cognitive symptoms. Thus, aberrant accumulation of amyloid and NFTs might indicate that these individuals are at a higher risk of developing AD [28, 81, 82].

We also investigated AD heterogeneity across different stages of disease severity by splitting the sample into amyloid positive and negative [83, 84]. For amyloid positive, results mimicked the full sample, albeit with a higher level of cognitive and brain deficits. For amyloid negative, the multilayer model identified three communities with a stepwise decrease in behavioral performance and an increase in brain pathology. Specifically, for most cognitive assessments, community 2 showed an intermediate position so that its subjects showed worse scores compared to community 1 (i.e., “healthiest” community) but better than those in community 3 (i.e., displaying most signs of “AD-related aging”). Exceptions to these were MMSE and CDRBSB, where the scores of community 2 were identical to those in community 1. The same occurred for volumetric brain markers, where community 2 was identical to community 1 in hippocampal volume. A deviation from this pattern was seen for CSF tau and amyloid PET, where the “intermediate” community 2 showed lower values than both community 1 and 3. This appears to indicate that those in community 3 have an objectively higher degree of deficits in most measurements, while those in community 2 might be part of a transitional state where there is not a high level of damage yet. Thus, this could explain why they show equivalent scores to community 1 in some measures but worse in others, while still having some CSF and PET markers within normal range [24, 71, 85, 86]. Perhaps subjects in community 2 are those for which interventions can work best. These findings, together with how the multilayer network separated early and late MCI across the full sample, might help answer questions such as “who is more likely to benefit from cognitive training or pharmacological interventions?”

Limitations of this study include the lack of individuals with different kinds of neurodegenerative disorders. Future work is necessary to test how the proposed multilayer network framework performs in distinguishing frontotemporal, mixed, and vascular dementia, or Parkinson’s disease, among others. Further, the AD and MCI diagnoses in our study were based on clinical consensus. AD can only be diagnosed with certainty after death and, although ADNI has a subsample with postmortem data and neuropathologic diagnosis, none of the current participants have this information. Finally, we acknowledge that there is inherent circularity in using cognition as a layer of our model, and therefore tried to circumvent this issue by not adding tests that were directly used for AD characterization in this layer. However, despite our efforts, these measures are still strongly correlated with those used for diagnostic purposes (e.g., MMSE and FAQ r = −0.61 *p* < 0.001). This might reduce interest in the model’s ability to differentiate between CN and AD but it is an intrinsic limitation of including cognition as part of a multilayer framework focused on identifying and predicting AD.

AD cannot be captured by any single modality due to its heterogeneity and multifactorial nature. Our study includes a myriad of different biomarkers and accounts for possible relationships among them, which is an advancement compared to previous research. This is because, although some markers have larger contributions than others for detecting AD and its progression, these markers are typically detached from each other [19, 25–27, 87]. We acknowledge that not all modalities have equal contributions, but they might relate to each other in complex ways. Our findings show that the multilayer framework is successful in capturing the complex relationship between different AD biomarkers and provides insight into the heterogeneity of the disease by identifying groups of intermediate and advanced levels of neurological and behavioral deficits. The fact that the model assigns a select number of CN participants to AD-dominant communities with concomitant volumetric decrease and Aβ and tau deposition further confirms this idea. By showing that considering relationships within and across modalities results in highly accurate predictions, our work indicates that AD is a good example of the well-known axiom “the whole is greater than the sum of its parts”.

### Supplementary information

Supplementary information

## Data Availability

Data used in the preparation of this article were obtained from the ADNI database (adni.loni.usc.edu). As such, although the investigators for ADNI contributed to the design and implementation of our study through their work for ADNI and/or provided data, they did not participate in the analysis or writing of this report.
