# Supplementary material for: Decoding the heterogeneity of Alzheimer’s disease diagnosis and progression using multilayer networks
Source: Mol Psychiatry. 2022 Dec 20;28(6):2423–32. doi: 10.1038/s41380-022-01886-z (PMC10279806; doi:10.1038/s41380-022-01886-z)
Supplement: Supplementary file 1 — Supplementary information [file 41380_2022_1886_MOESM1_ESM.docx]

**Supplementary information for**

Decoding the heterogeneity of Alzheimer’s disease diagnosis and progression using multilayer networks

Bárbara Avelar-Pereira^1*^, Michael E. Belloy^2^, Ruth O’Hara^1^, and S. M. Hadi Hosseini^1*^ for the Alzheimer’s Disease Neuroimaging Initiative

^1^Department of Psychiatry and Behavioral Sciences, School of Medicine, Stanford University, Stanford, CA 94304, USA

^2^Department of Neurology and Neurological Sciences, School of Medicine, Stanford University, Stanford, CA 94304, USA

***To whom correspondence may be addressed**. Bárbara Avelar-Pereira and S. M. Hadi Hosseini, Department of Psychiatry and Behavioral Sciences, School of Medicine, Stanford University, Stanford, CA 94304, USA. Phone number: 650-679-3985. Email addresses: [barave@stanford.edu](mailto:barave@stanford.edu) and [hosseiny@stanford.edu](mailto:hosseiny@stanford.edu).

This PDF contains Supplementary text, figures S1-S10, and Table S3-1, S3-2, and S7-1.

**S1**: **Structural imaging and Amyloid-β PET features**


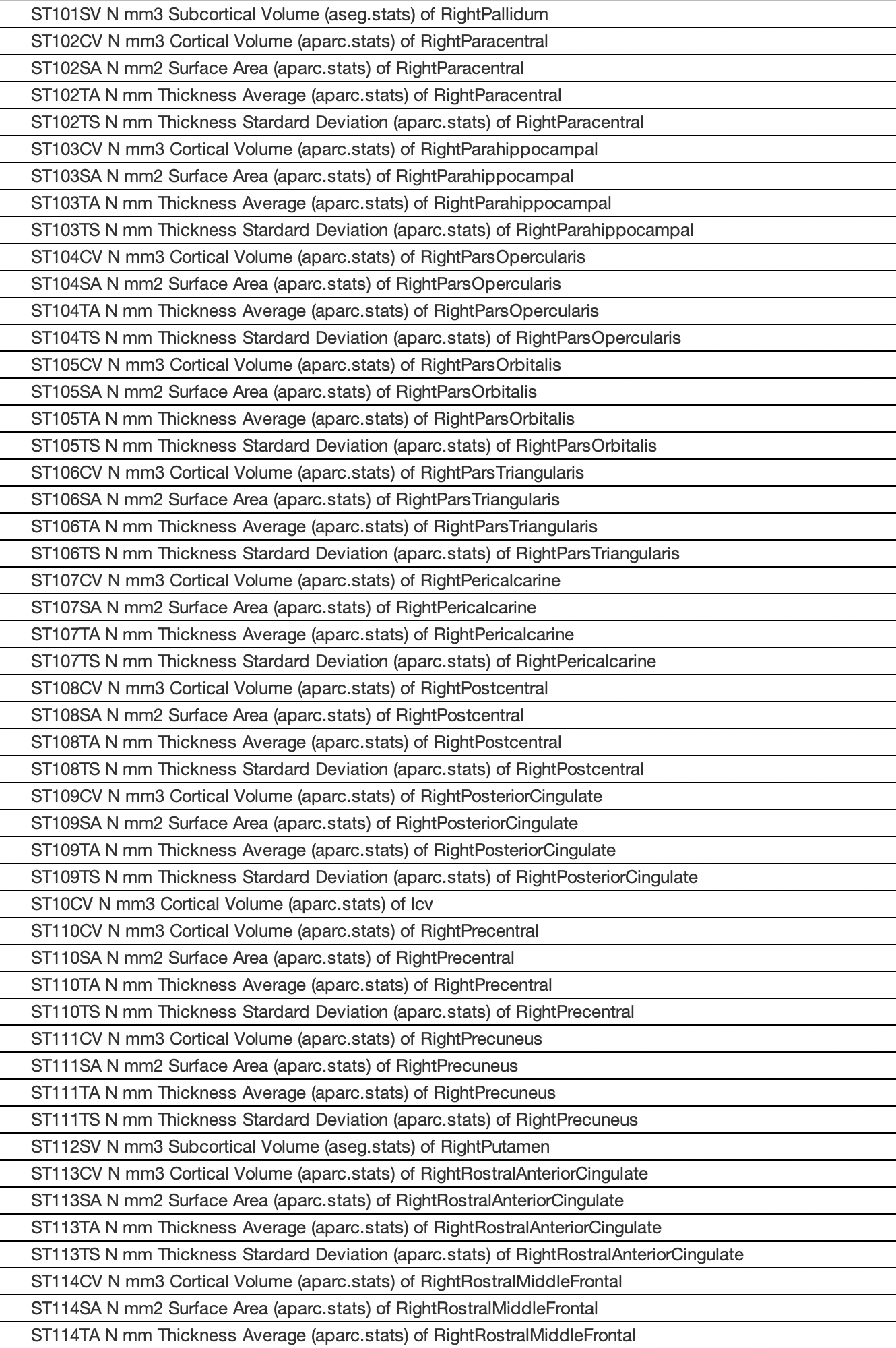


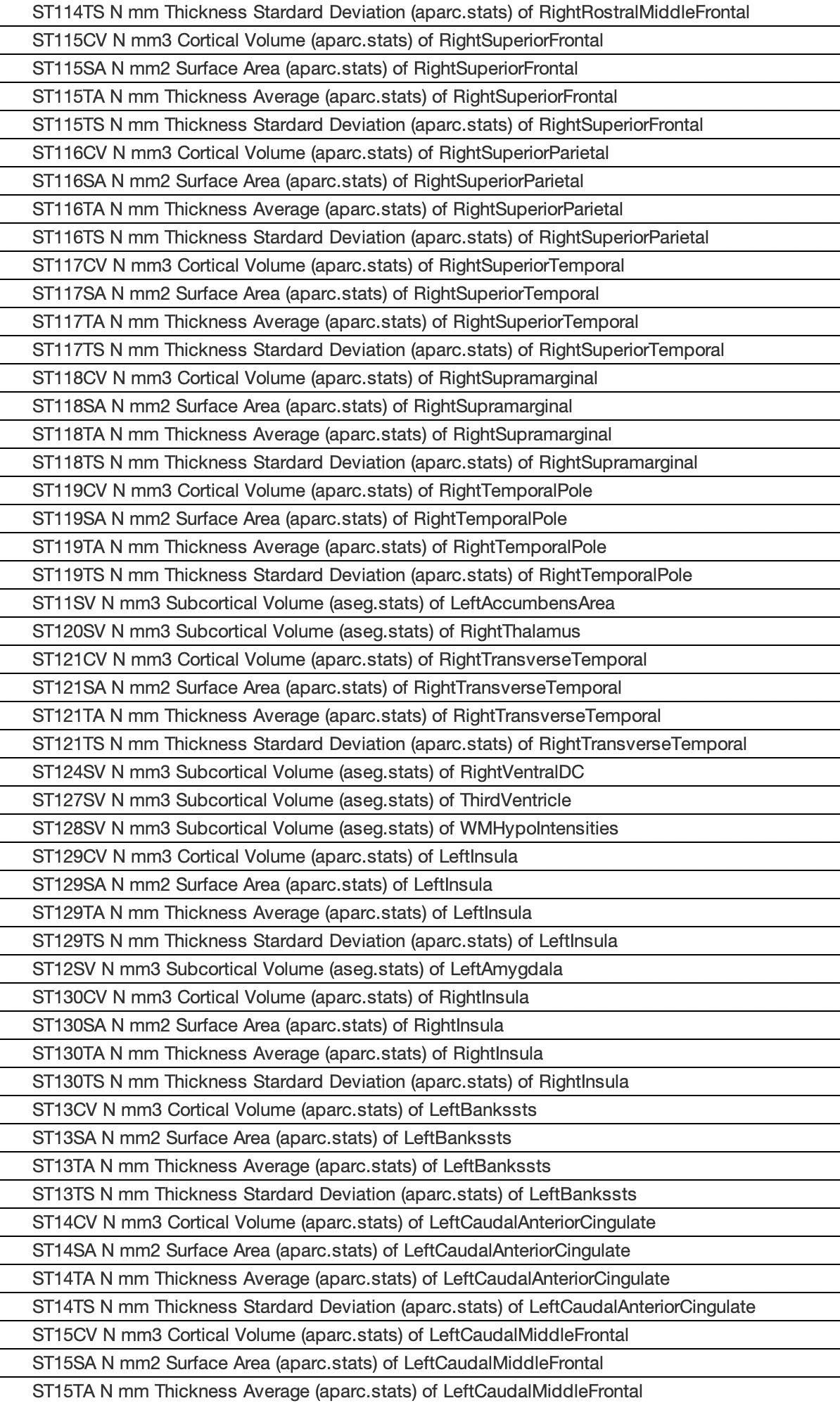


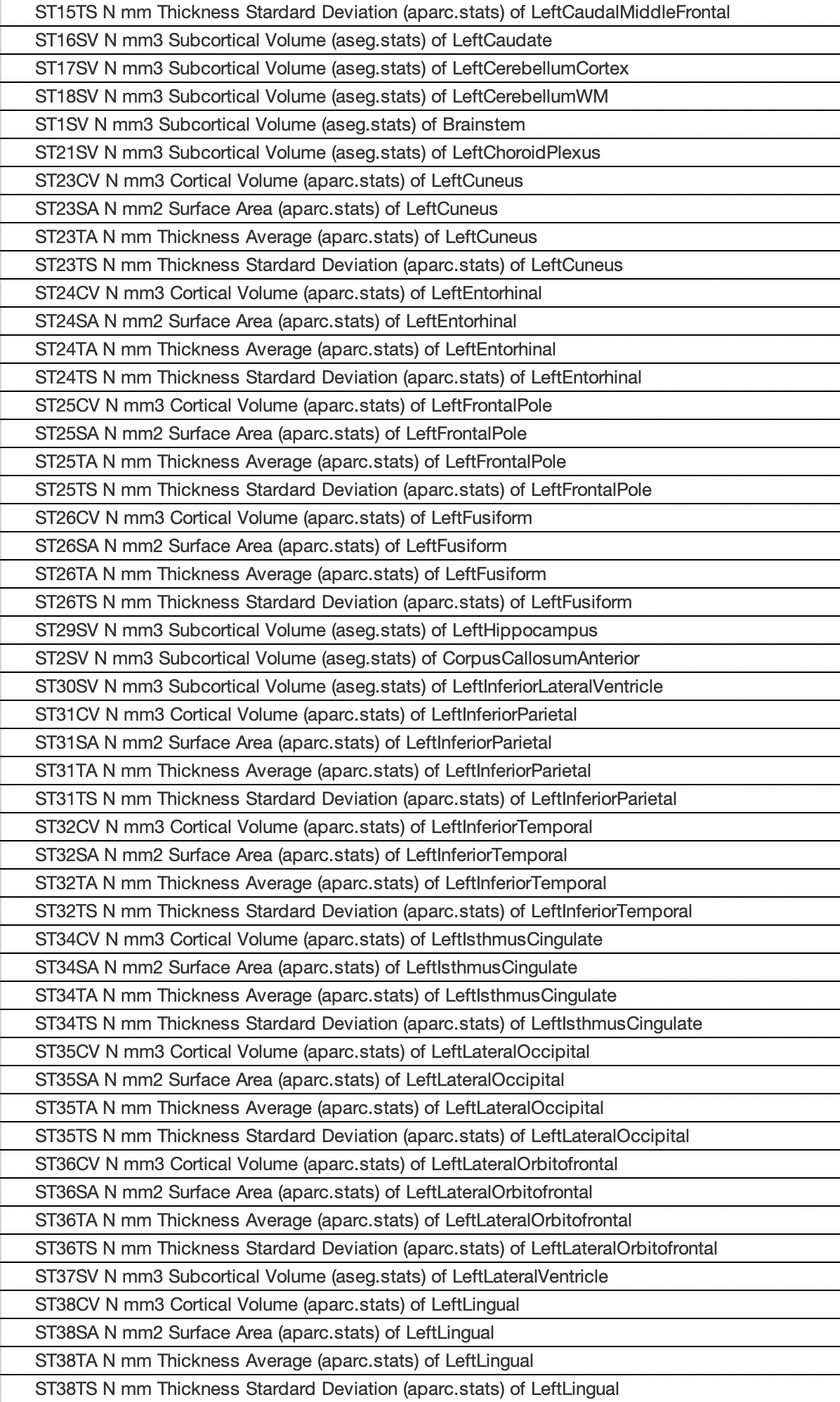


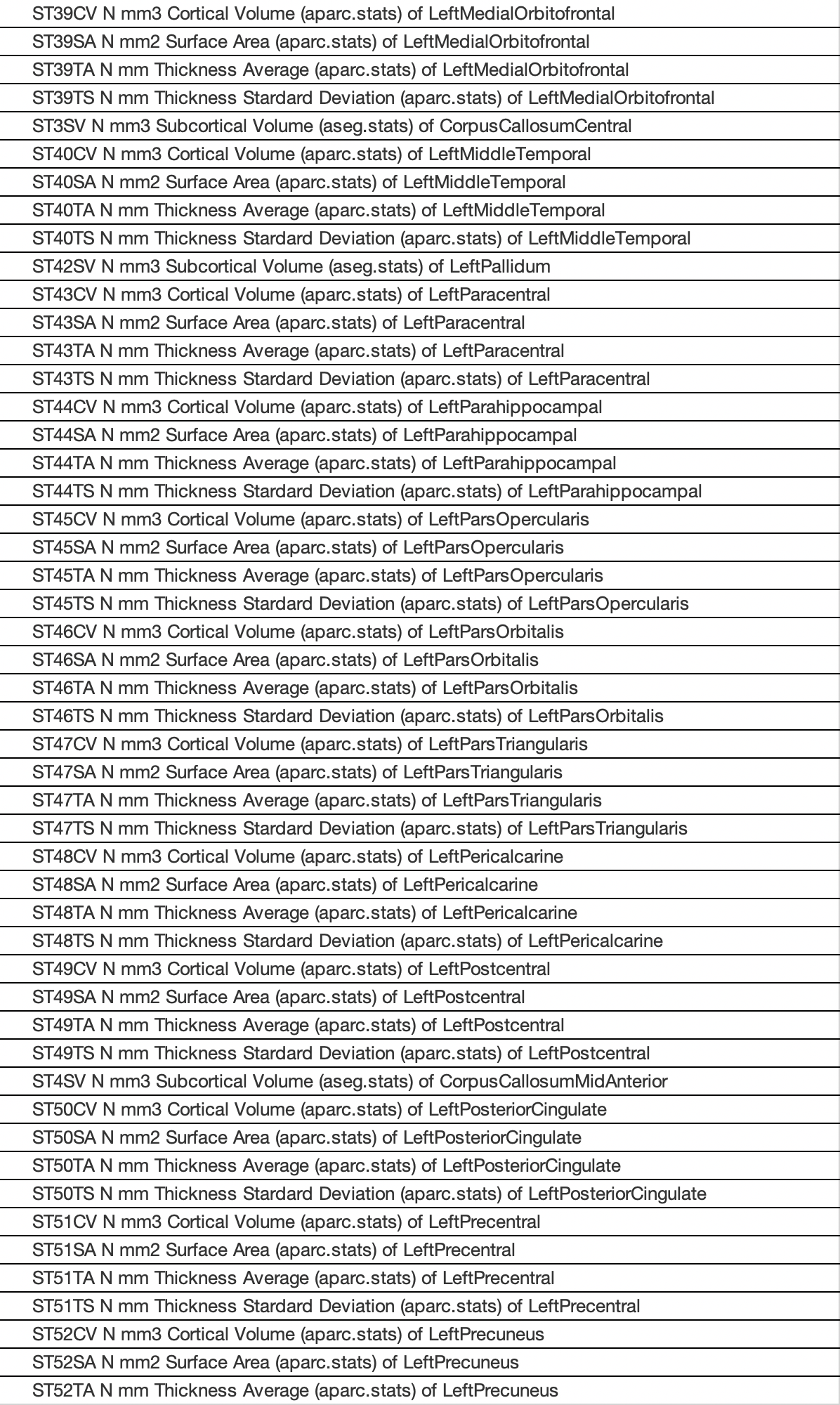


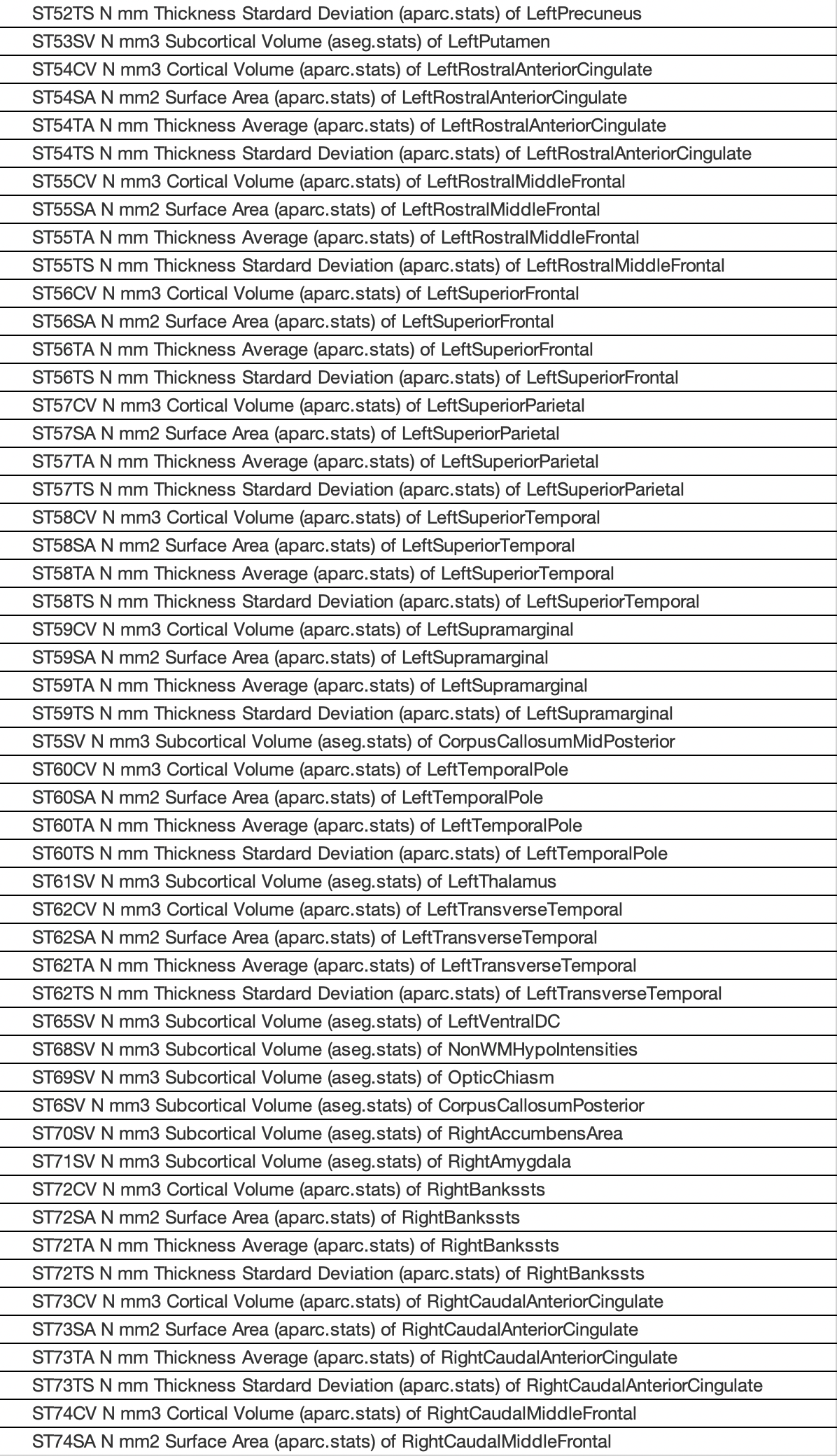


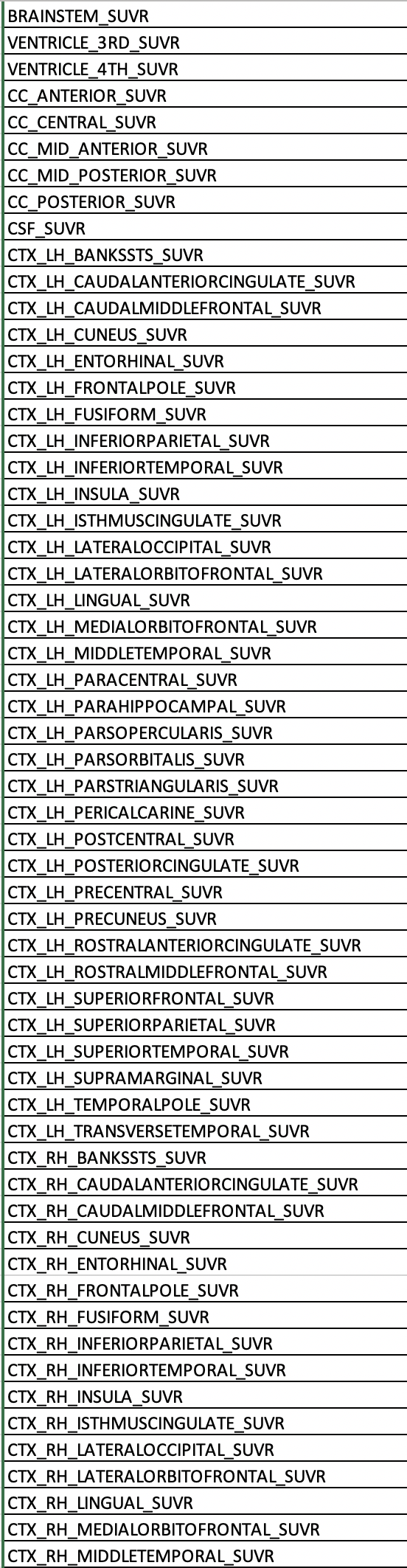

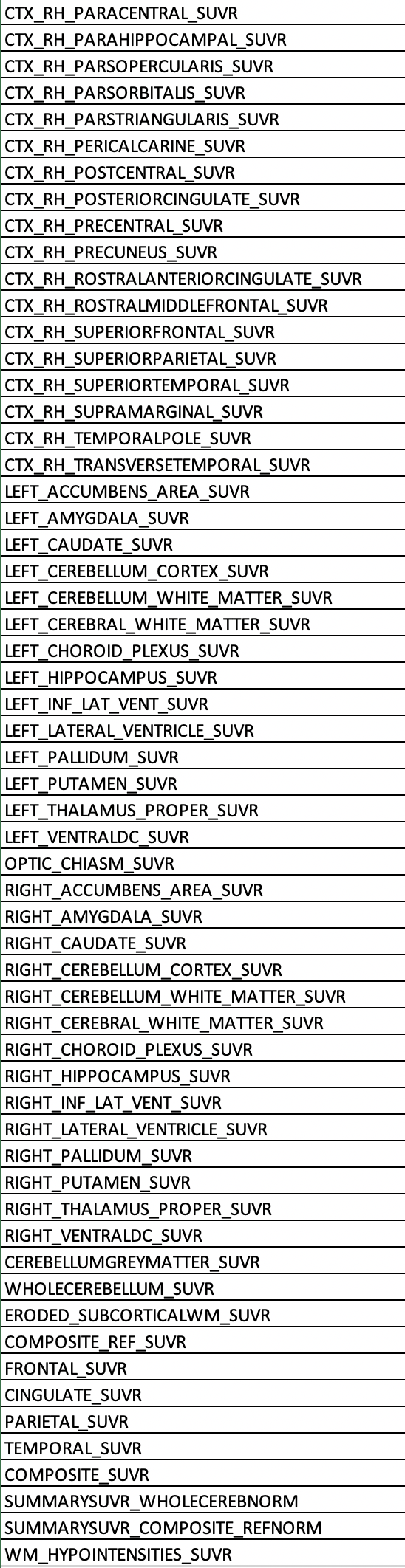


**S2: Multilayer networks**

Multilayer networks maximize modularity in a similar way to that of other networks. The partition of nodes into modules is optimized by maximizing a modularity-quality function that compares edge weights in a network to expected edge weights in a null network. Thus, the iterative generalized Louvain (GenLouvain) community detection algorithm detects communities robust to null models. The modularity quality function (*Q*) is defined as:

$$Q =\frac{1}{2\mu}\sum_{ijlr} \{\left( A_{ijl}- \gamma_{l}M_{ijl} \right)\delta_{lr}+\delta_{ij}\omega_{jlr}\}(\delta\left( g_{il},g_{jr} \right))$$

where the matrix of layer *l* has components $A_{ijl}$ and $M_{ijl}$ gives the corresponding components for the optimization null model. The structural resolution or scaling parameter ($\gamma$) of layer *l* is $\gamma_{l}$, $g_{il}$ and $g_{jr}$ are the community assignments of node *i* in layer *l* and node *j* in layer *r*, $\omega_{jlr}$ corresponds to the interlayer coupling strength parameter connecting node *j* in layers *l* and *r*. Finally, $\mu$ is the total edge weight in the network.

We used the null model formulation by Mucha et al. (2010), where authors generalized the equivalence between the modularity-quality function and communities’ stability under Laplacian dynamics to reproduce the null models for bipartite, directed, and signed networks. These generalizations were then applied to derive null models for multilayer networks that extend the quality-function methodology and include an additional parameter ($\omega$) to control the coupling between layers. Briefly, this extension of single-layer modularity to multilayer networks measures the stability of a community by comparing the probability of a random walker to stay in the same community at time 𝑡 to the static solution (i.e., 𝑡→∞). Each layer is described by its adjacency matrix and includes interlayer couplings which connect a node (i.e., subject) to itself in another layer. This coupling strength is usually taken to be 0 or a constant $\omega$, indicating absence or presence of inter-layer links. There are no pre-established values for either $\omega$ (interlayer coupling) or $\gamma$ (structural scaling parameter). Increasing $\gamma$ results in a more fragmented structure, so we attempted several thresholds ($\gamma$ = 0.2, 0.4, 0.5, …1) with results being fairly consistent (e.g., two large communities, with accuracies of between 75-90% for AD detection, 70-92% for MCI to CN reverters, and 75-91% for MCI to AD converters). We report here the results that give the best demarcation between communities ($\gamma$ = 0.2). We considered a range = 0, 0.1, 1 for $\omega$ and report results for $\omega$ = 1.

**S3**: **Communities for individual modalities (full sample)**

In this section, we report the communities obtained from each modality individually (for an overview see Supplementary Figure 1). In summary, and similarly to what we observed in the multilayer network, the amyloid, CSF, and genetics layers consisted of two communities. For amyloid, community 1 included 82% of all CN individuals and community 2 had 88.1% of all Alzheimer’s disease cases. MCI participants were mostly located in community 1 (60.3%), together with those considered CN, suggesting that PET is capable of capturing individuals’ Alzheimer’s disease pathology but only after reaching a given amyloid threshold. For CSF, community 1 had 62% of CN controls and community 2 had 90.4% of Alzheimer’s disease cases. However, MCI was more or less equally split between the two groups (57.8% vs. 42.2%). The genetics layer resulted in both CN and Alzheimer’s disease being evenly divided between communities (45.3% vs. 54.7% for CN; 48.1% vs. 51.9% for Alzheimer’s). A similar trend was seen for MCI, with community 1 including 53.4% of cases and community 2 including 45.7%. In regard to cognition, most healthy participants were part of community 3 (81.3%) and most of the Alzheimer’s disease cases was part of community 1 (91.1%). Even though the Alzheimer’s disease number is identical to that identified by the multilayer network, it is noteworthy that the cognitive layer also included an additional community (i.e., community 2) with only two individuals whose affiliation seemed equally strong to either of the other two groups. One of these one was CN and the other was an MCI subject. Finally, the volumetric modality resulted in three communities as well. Here, CN individuals were mostly part of community 2 (52.7%) but there was also a sizable number of subjects in community 1 (32%) and 3 (20.6%). For Alzheimer’s disease, 42.2% of cases were in community 3, but they were also present in community 1 (32.6%) and 2 (25.2%). The same trend was seen for MCI, where 49.2% of cases were part of community 2, but community 1 and 3 still included a good portion of the sample (30.2% vs. 20.6%).


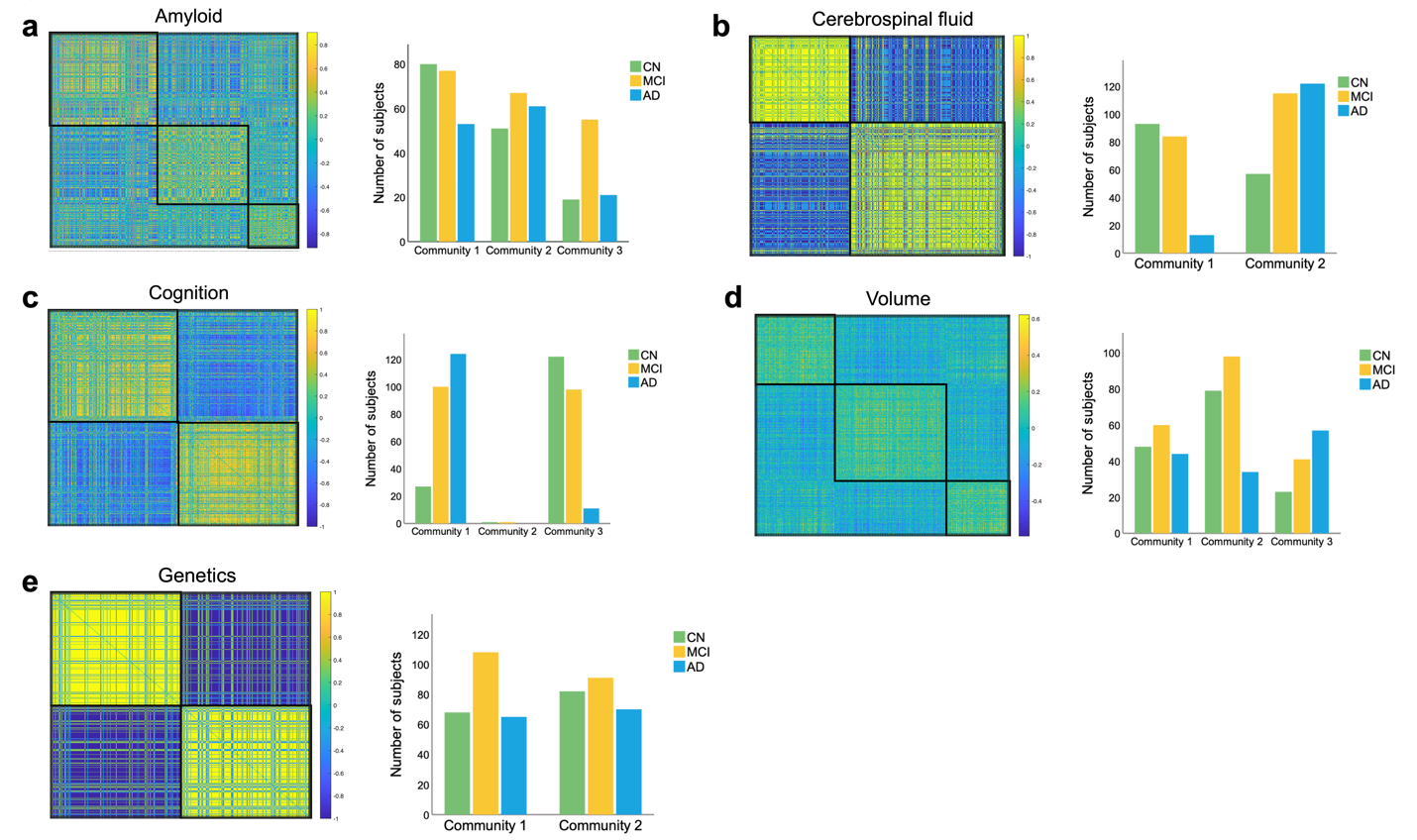


**Fig. S3.** Monolayer networks and respective sample distribution in each diagnosis group for **(a)** amyloid-β PET, **(b)** CSF, **(c)** cognition, **(d)** FreeSurfer volumetric estimates, and **(e)** genetics.

**S4: Comparison of stable MCI cases between communities (full sample)**

We compared stable MCI subjects across communities to analyze whether there were cognitive or biological differences between them. In summary, we found that although they had the same age (*t* = -1.52, *p* = 0.13), those in community 2 had lower MMSE (*t* = 3.62, *p* = 0.001), worse ADAS13 scores (*t* = -3.339, *p* = 0.001) and memory (*t* = 3.98, *p* < 0.001). Years of education (*t* = 2.034, *p* = 0.044), executive functioning (*t* = 1.95, *p* = 0.05) and hippocampal volume (*t* = 1.94, *p* = 0.05) were not statistically different, as they did not survive Bonferroni correction. Their CDRSB (*t* = -0.68, *p* = 0.5) and MoCA scores (*t* = 1.637, *p* = 0.116) were also comparable and no differences were found in the entorhinal cortex (*t* = 1.329, *p* = 0.186) or in whole brain volume (*t* = 0.874, *p* = 0.383). Still, community 2 had more individuals with one or two ε4 allele(s) (χ(4) = 40.088, *p* < 0.001) and higher tau (*t* = -8.47, *p* < 0.001), pTau (*t* = - 8.62, *p* < 0.001), and lower amyloid-β (*t* = 13.36, *p* < 0.001) as measured by CSF. Importantly, subjects in community 2 also had higher PET amyloid-β (*t* = -17.656, *p* < 0.001).

**
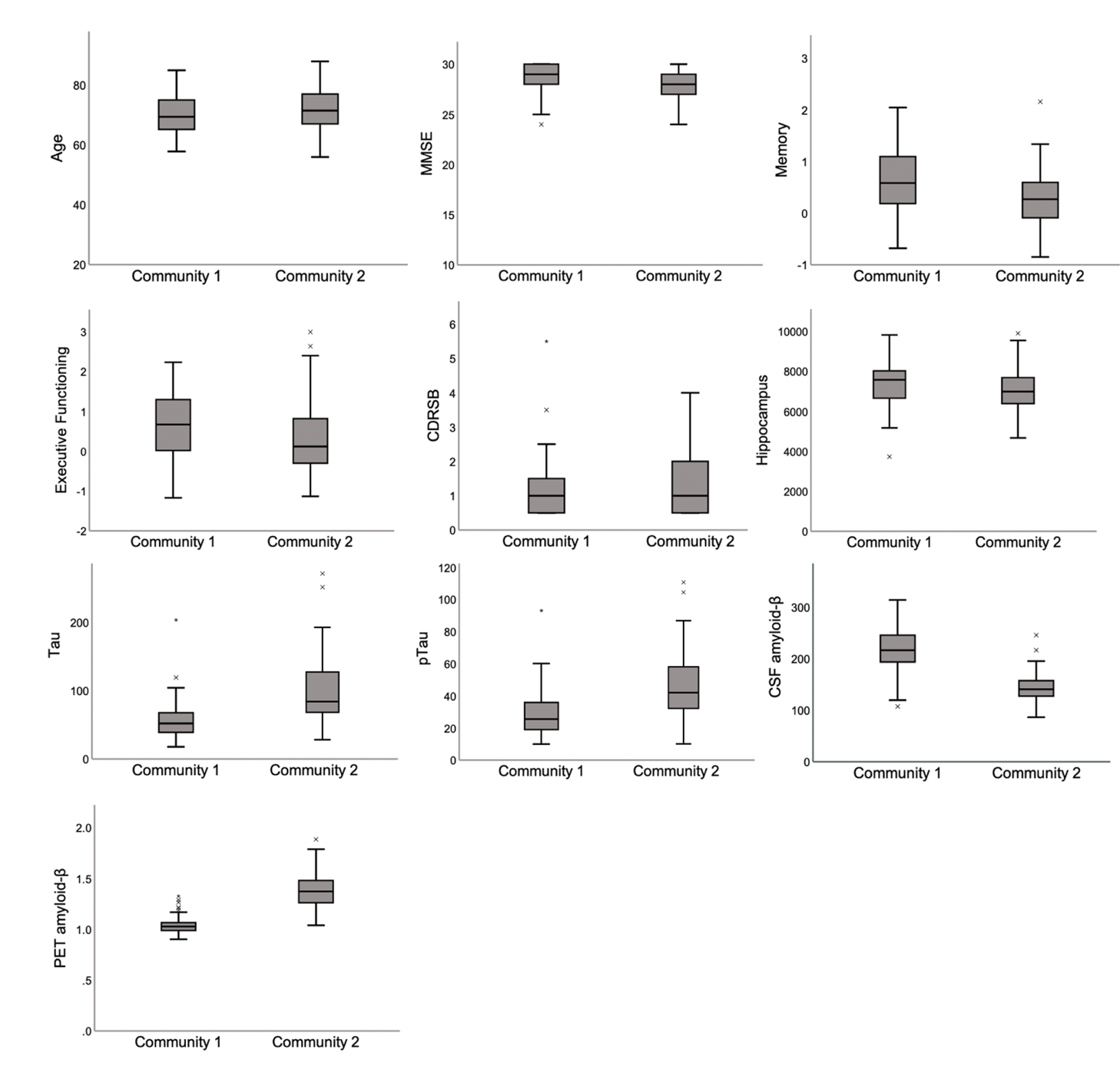
Fig. S4.** Boxplots for stable MCI subjects in community 1 and 2 for age, MMSE, memory, executive functioning, CDRSB, hippocampal volume, CSF tau, pTau, amyloid-β, and PET amyloid-β.

**S5: Mismatched Alzheimer’s disease cases (i.e., Alzheimer’s disease cases grouped with the CN dominant community)**

| S5 - Table 1. Multilayer communities | | |
| --- | --- | --- |
|  | **AD in community 1 (N = 12)** | **AD in community 2 (N = 123)** |
| **Age** | 74.7 ± 8.30 | 72.88 ± 25.61 |
| **Years of education** | 14.9 ± 2.7 | 17.1 ± 2.59 |
| **MMSE** | 26.67 ± 2.99 ↑ | 25.61 ± 2.69 |
| **ADAS13** | 15.3 ± 8.8 | 15.0 ± 7.79 |
| **MoCA** | 20.08 ± 4.72 | 20.23 ± 3.72 |
| **Memory** | -0.06 ± 0.65 ↑ | -0.49 ± 0.63 |
| **Executive functioning** | -0.079 ± 0.80 ↑ | 3.19 ± 0.93 |
| **CDRSB** | 2.5 ± 1.65 ↑ | 3.18 ± 1.83 |
| **Hippocampal volume** | 6832.91 ± 1305.5 ↑ | 6147.12 ± 1051.00 |
| **Entorhinal volume** | 3411.5 ± 907.92 ↑ | 3136.9 ± 788.99 |
| **Whole brain volume** | 1043636.9 ± 1261159.3 ↑ | 1033818.48 ± 115783.423 |
| **CSF Tau** | **76.375 ± 39.20 ↑*** | 133.00 ± 56.94 |
| **CSF pTau** | **35.85 ± 13.49 ↑*** | 59.28 ± 22.76 |
| **CSF amyloid-β** | **215.42 ± 131.38 ↑*** | 131.38 ± 23.38 |
| **PET amyloid-β** | **1.02 ± 0.03 ↑*** | 1.42 **±** 0.01 |

**↑** Indicates those for which Alzheimer’s disease subjects in community 1 had better mean outcomes.

* Indicates those for which there were significant differences between groups (p ≤ 0.001).

**
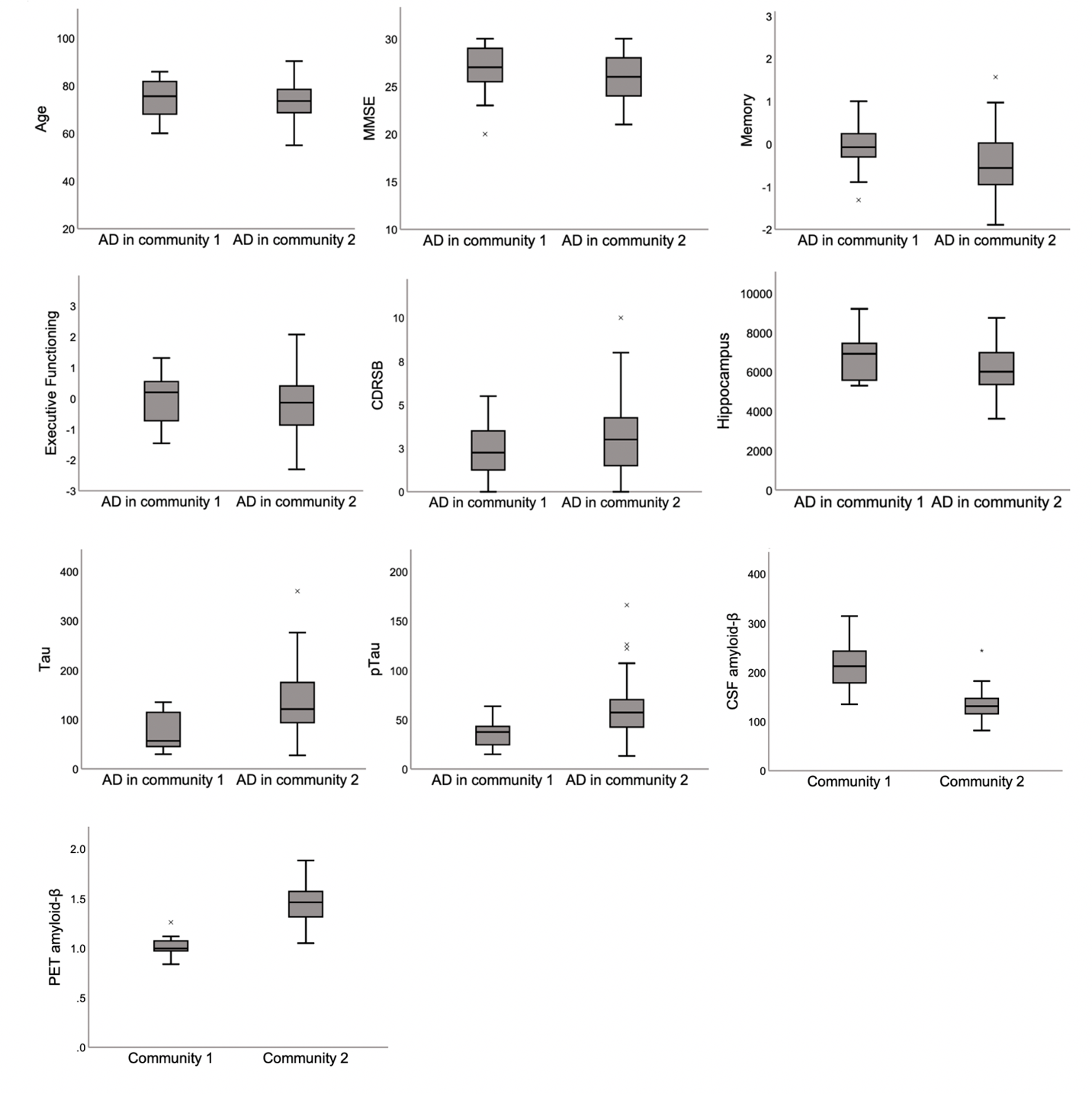
**

**Fig. S5.** Boxplots for Alzheimer’s disease subjects in community 1 and 2 for age, MMSE, memory, executive functioning, CDRSB, hippocampal volume, CSF tau, pTau, amyloid-β, and PET amyloid-β.

**Mismatched CN cases (i.e., CN cases grouped with the Alzheimer’s disease dominant community)**

| S5 - Table 2. Multilayer communities | | |
| --- | --- | --- |
|  | **CN in community 1 (N = 123)** | **CN in community 2 (N = 27)** |
| **Age** | 71.03 ± 6.40 | 73.31 ± 6.08 ↑ |
| **Years of education** | 16.80 ± 2.57 | 16.59 ± 2.05 ↑ |
| **MMSE** | 29.14 ± 1.15 | 29.04 ± 0.85 ↑ |
| **ADAS13** | 8.358 ± 4.116 | 9.2593 ± 4.337 ↑ |
| **MoCA** | 26.18 ± 2.31 | 25.89 ± 1.99 ↑ |
| **Memory** | 1.18 ± 0.57 | 0.95 ± 0.56 ↑ |
| **Executive functioning** | 1.18 ± 0.81 | 0.69 ± 0.67 ↑ |
| **CDRSB** | 0.22 ± 0.59 | 0.241 ± 0.59 ↑ |
| **Hippocampal volume** | 7693 ± 846.9 | 7238 ± 934.5 ↑ |
| **Entorhinal volume** | 3969 ± 582.94 | 3730 ± 400.46 ↑ |
| **Whole brain volume** | 1069570.54 ± 103343.3 | 997321.1 ± 86709.3 ↑ |
| **CSF Tau** | 56.61 ± 20.77 | **82.94 ± 37.15 ↑*** |
| **CSF pTau** | 28.49 ± 12.27 | **42.73 ± 17.72 ↑*** |
| **CSF amyloid-β** | 216.4 ± 40.78 | **146.5 ± 23.70 ↑*** |
| **PET amyloid-β** | 1.03 **±** 0.07 | **1.35 ± 1.89 ↑*** |

**↑** Indicates those for which CN subjects in community 2 had worse mean outcomes.

* Indicates those for which there were significant differences between groups (p ≤ 0.001).

**
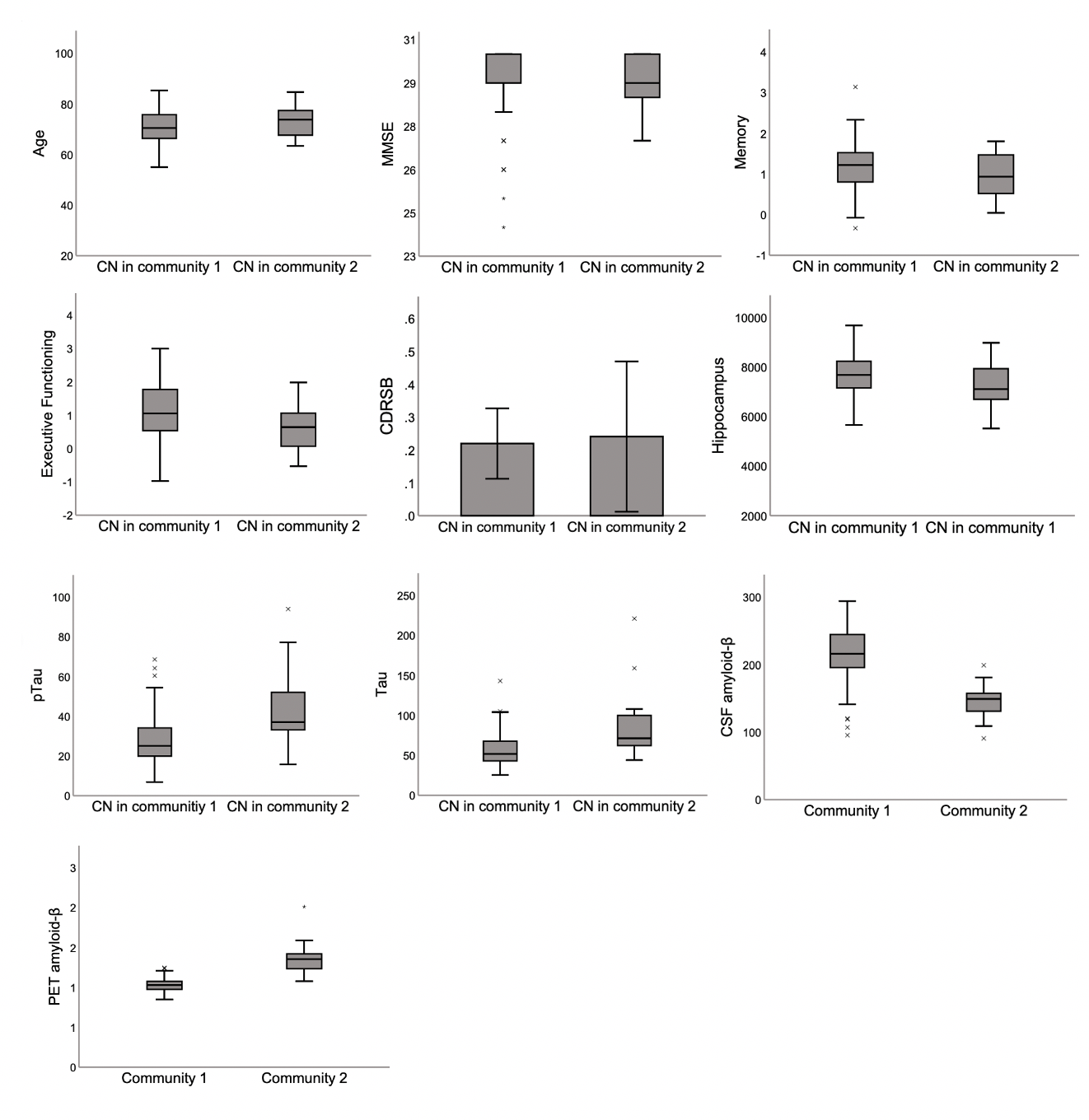
**

**Fig. S5-2.** Box and bar plots for CN subjects in community 1 and 2 for age, MMSE, memory, executive functioning, CDRSB, hippocampal volume, CSF tau, pTau, amyloid-β, and PET amyloid-β.

**S6: Validation**

We validated our findings by splitting the original sample chronologically into three equal parts. The first two-thirds were used to compute a multilayer network and the resulting communities were compared to the one-third sample using participants’ cognitive scores (i.e., CDRSB, ADAS13, MMSE, MoCA), amyloid-β PET, CSF tau, pTau, and amyloid-β, and hippocampal, entorhinal, and whole brain volumes. We computed the similarity between each and every subject in the testing set (i.e., one-third group) with all subjects in the training set (i.e., two-thirds group), with results being converted into z-scores. Then, for each subject *K* in the testing set, we averaged the standardized similarity scores (z-scores) between subject *K* with each and every subject in community 1 (from the training set) and compared those with the average similarity between subject *K* and each and every subject in community 2 (also from the training set). If a subject was more similar on average to community 1 than community 2, they were allocated to that community and vice versa. This way, we were able to build the model using older data and tested it on newly collected data (i.e., testing it on new subjects as they become available).

The validation analysis confirmed results obtained from the full sample. Specifically, 91.2% of Alzheimer’s disease subjects at baseline were assigned to community 2 (the Alzheimer’s disease dominant community in the training set), whereas 78.9% of healthy individuals in the testing set were assigned to community 1 (the CN dominant community in the training set). MCI cases were again split between community 1 (46.3%) and community 2 (53.7%). However, the patterns observed in the full sample analyses were still apparent, where most EMCI at baseline were part of community 1 (61.3%) and most LMCI were part of community 2 (66.7%). Of note, the sample size is much smaller (1/3 of the original full sample) and the testing set was computed using the most newly acquired data, which leads to less time for longitudinal effects to occur.

**
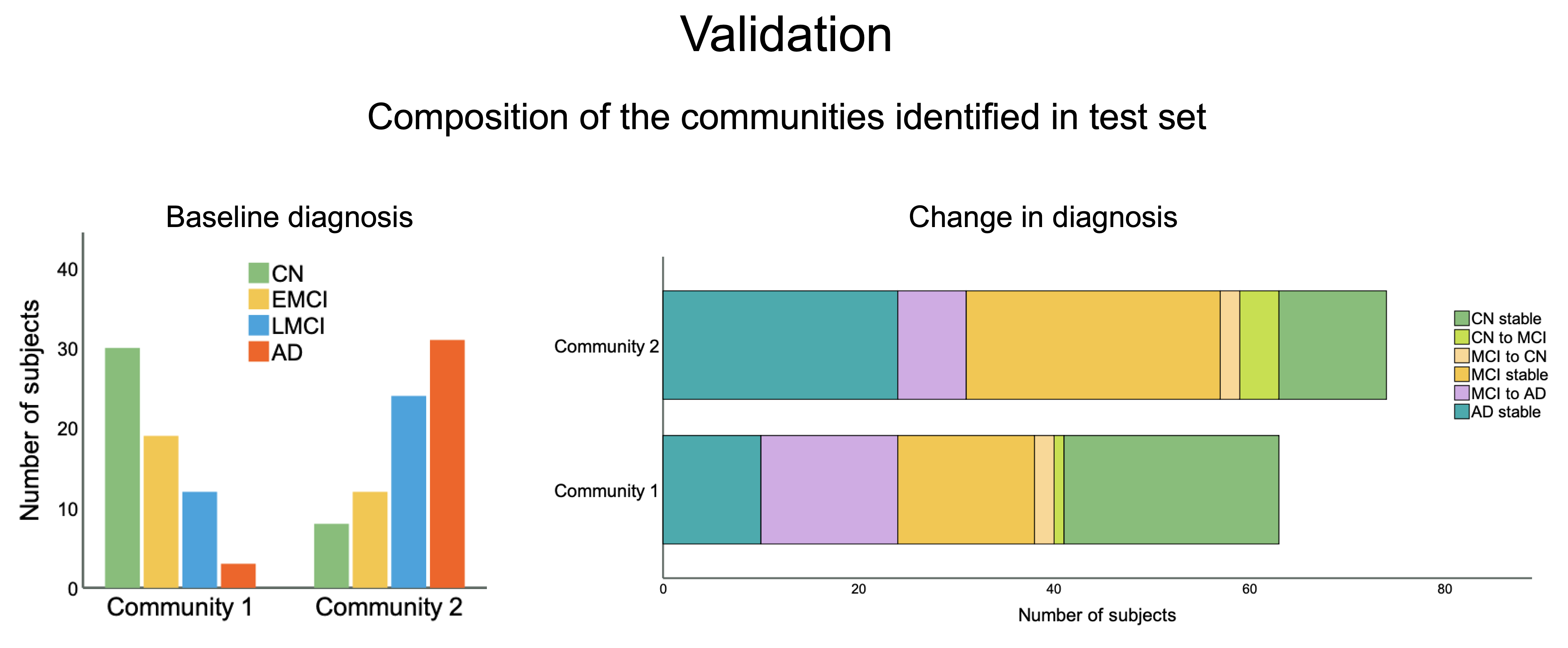
**

**Fig. S6.** Results of the validation analysis. On the left is the community allocation and sample distribution per diagnosis group and on the right are the communities divided by change in diagnosis for individuals in the one-third testing sample.

**S7: Sensitivity, specificity, and accuracy over time for converters/reverters**

| S7 – Table 1. Sensitivity, specificity, and accuracy for individuals who progress to AD and those who regress to CN from an MCI status | | | |
| --- | --- | --- | --- |
| **Time** | **Sensitivity** | **Specificity** | **Accuracy** |
| 12 months | 92.31% | 100% | 94.29% |
| 24 months | 95.56% | 100% | 96.30% |
| 36 months | 95.62% | 93.75% | 96.55% |
| 48 months | 89.47% | 83.3% | 87.5% |
| Final diagnosis | 90.79% | 88.46% | 90.20% |


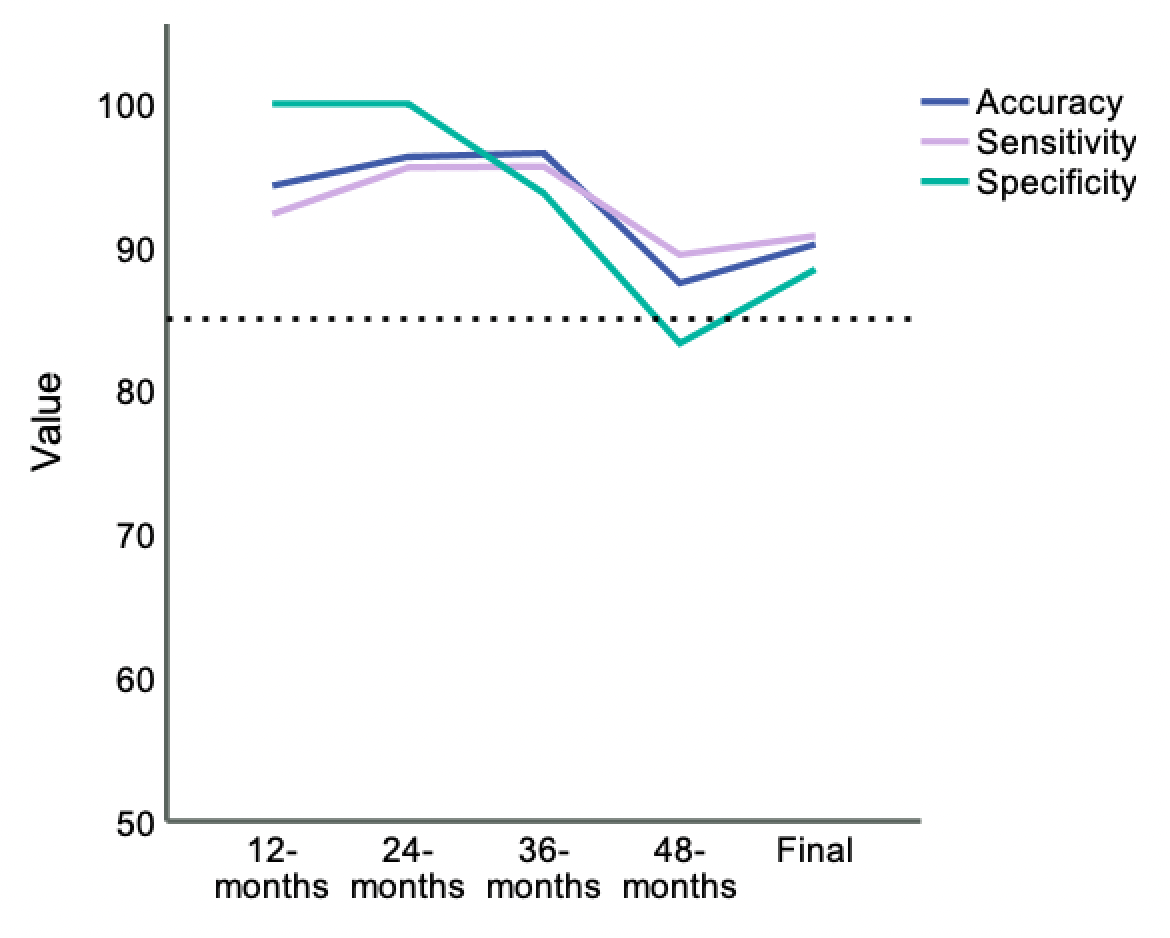


**Fig. S7**. Change in accuracy, sensitivity and specificity for MCI converters/reverters. The dotted line demonstrate that these analyses are above an 85% threshold.

**S8: Amyloid negative subsample**

**
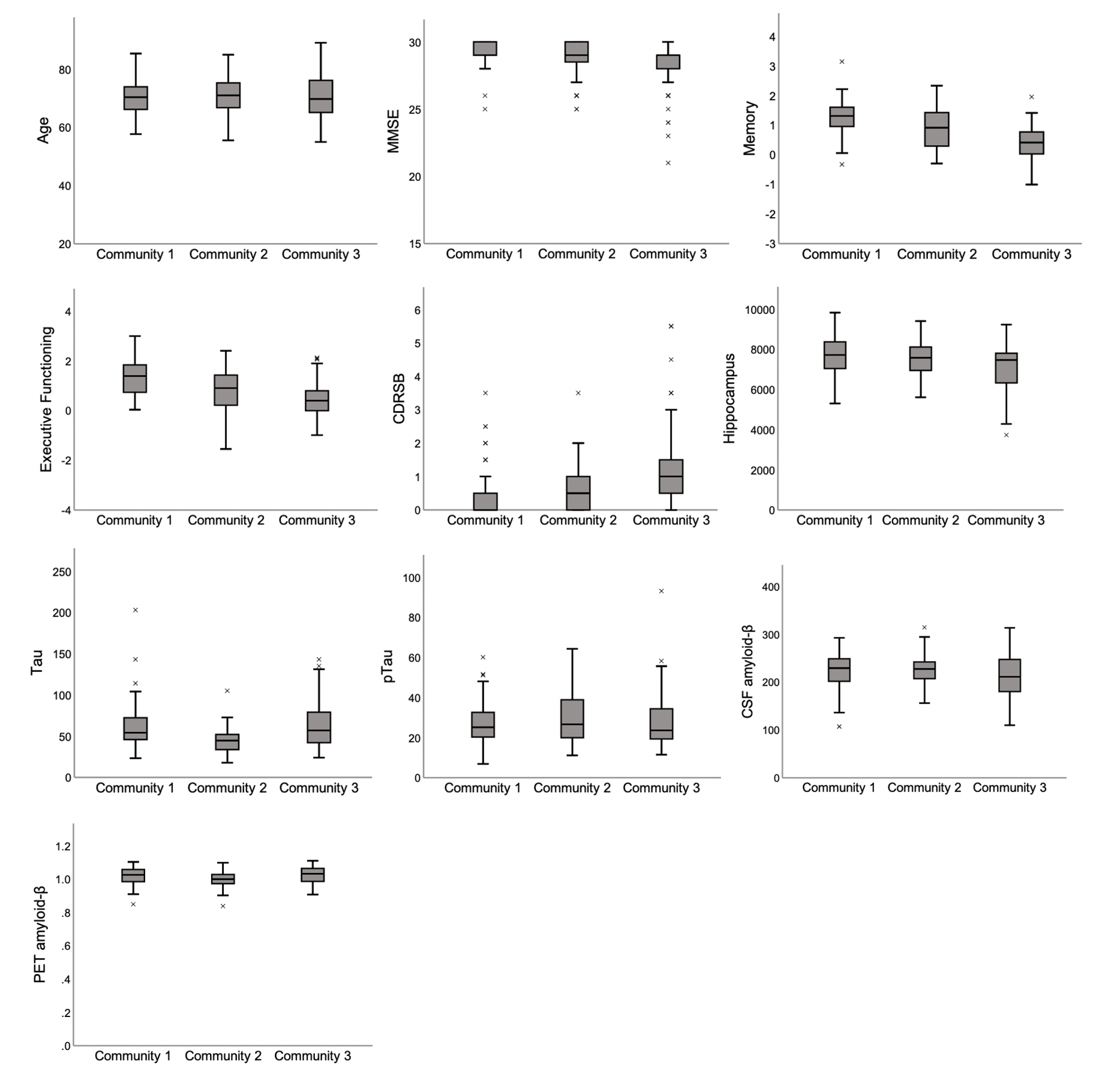
**

**Fig. S8.** Boxplots for amyloid negative subjects in community 1, 2, and 3, for age, MMSE, memory, executive functioning, CDRSB, hippocampal volume, CSF tau, pTau, amyloid-β, and PET amyloid-β.

**S9: Longitudinal amyloid negative and positive subsamples**


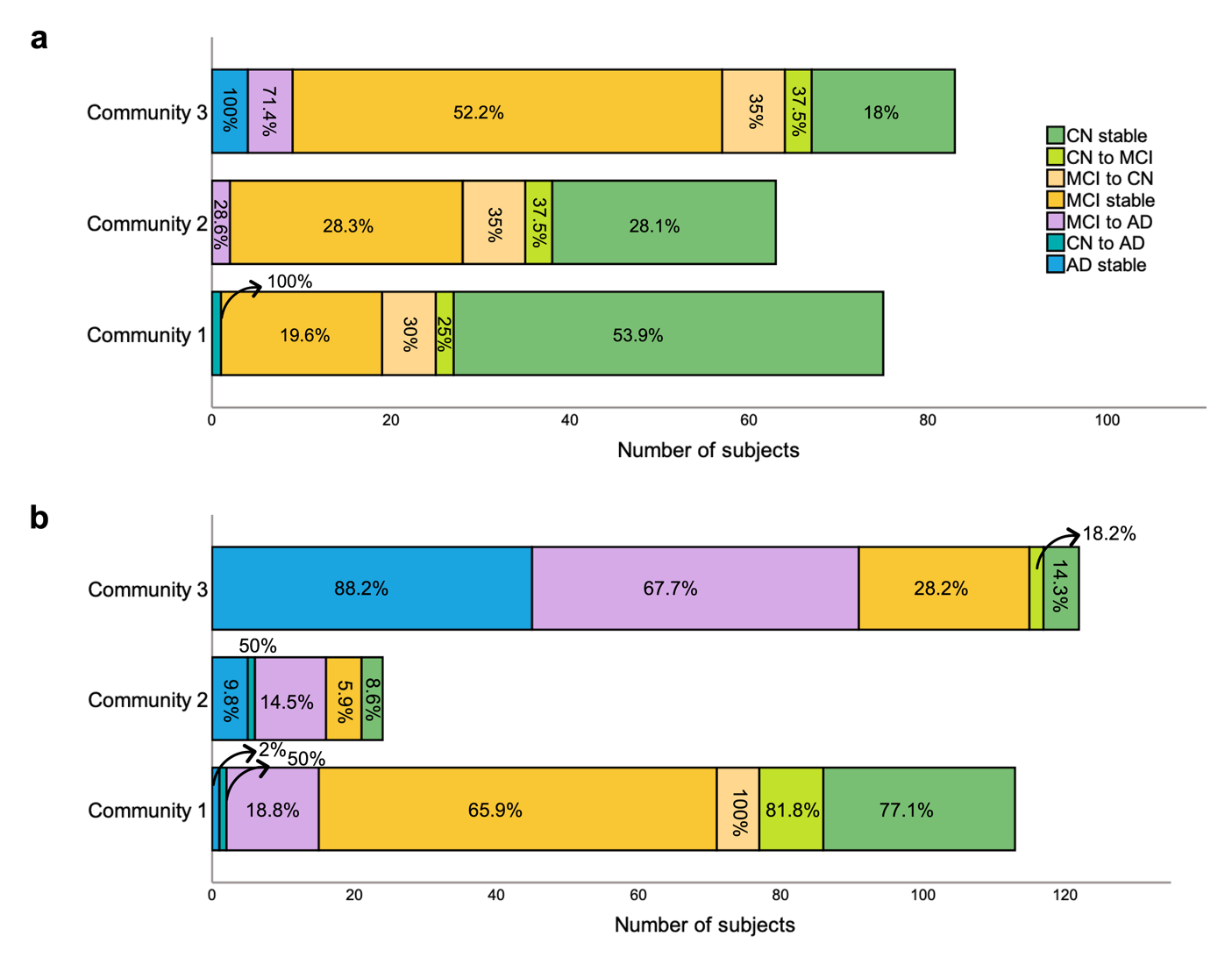


**Fig. S9.** Community 1, 2, and 3, divided by change in diagnosis for amyloid **(a)** negative and **(b)** positive subjects.

**Amyloid negative subsample**

Overall, the results suggest that community 1 corresponds to the healthiest group of subjects. It includes the largest number of CN individuals and, although it also comprises MCI cases, it only has 19.6% of those with a stable condition and the lowest number of participants who, at their final follow-up, progressed to MCI (25% compared to 37.5% in community 2 and 3). Moreover, only 30% of reverters are part of this community, compared to 35% in community 2 and 3. This is not a large difference as it seems those who regress to CN are equally spread-out across groups. However, it might also reflect the fact that community 1 has the lowest number of MCI subjects in general, with only 19.4% of all cases. There is one CN individual who, at their final follow-up, is presented as Alzheimer’s disease but it is important to point out that this community does not include any baseline Alzheimer’s disease dementia or MCI who go on to develop Alzheimer’s disease within the timeframe of the study. In second place, community 2 seems to reflect an intermediate stage, where many individuals have MCI, but some revert to CN (35%), some remain stable (28.3%), and others eventually develop Alzheimer’s disease (28.6%). Still, there are no baseline Alzheimer’s disease subjects in this community either. It also has less stable CN subjects than community 1 but more than community 3 (28.1%). Finally, the last community includes all Alzheimer’s disease individuals at baseline and the largest number of MCI cases that develop Alzheimer’s disease (71.4%). It also contains the largest percentage of stable MCI (52.2%) within the time frame of the study. Although it seems likely that part of these stable participants might be MCI cases who would later progress to AD, we cannot make any definite claims as this would require a larger follow-up interval. Community 3 also has the lowest number of stable healthy individuals (18%) and 37.5% of CN individuals who develop MCI. Even though it includes a good number of reverts (35%), these individuals could possibly represent unstable cases who change from CN to MCI at different timepoints.

**Amyloid positive subsample**

Here, community 3 includes the majority of individuals who had a stable Alzheimer’s disease diagnosis (88.2%) and the majority of MCI cases that progressed to Alzheimer’s disease (67.7%). It also includes 28.2% of those with stable MCI, but as it occurred in amyloid negative subjects, it is unclear whether given a larger follow-up they would have joined the group of MCIs who become Alzheimer’s disease cases. Community 3 included a small number of stable CN (14.3%) subjects and CN who become MCI patients (18.2%), with community 1 including most of both instances (stable CN: 77.1%; CN to MCI: 81.8%). Community 1 also had the largest number of stable MCI cases (65.9%) and, although all individuals are amyloid positive, it had a small number of individuals with Alzheimer’s disease be it those with MCI who progressed to Alzheimer’s disease (18.8%) or those with stable Alzheimer’s disease (2%). Finally, it included 1 subject who was CN at baseline but whose final diagnosis was of Alzheimer’s disease dementia.

**S10: Amyloid positive subsample**

**
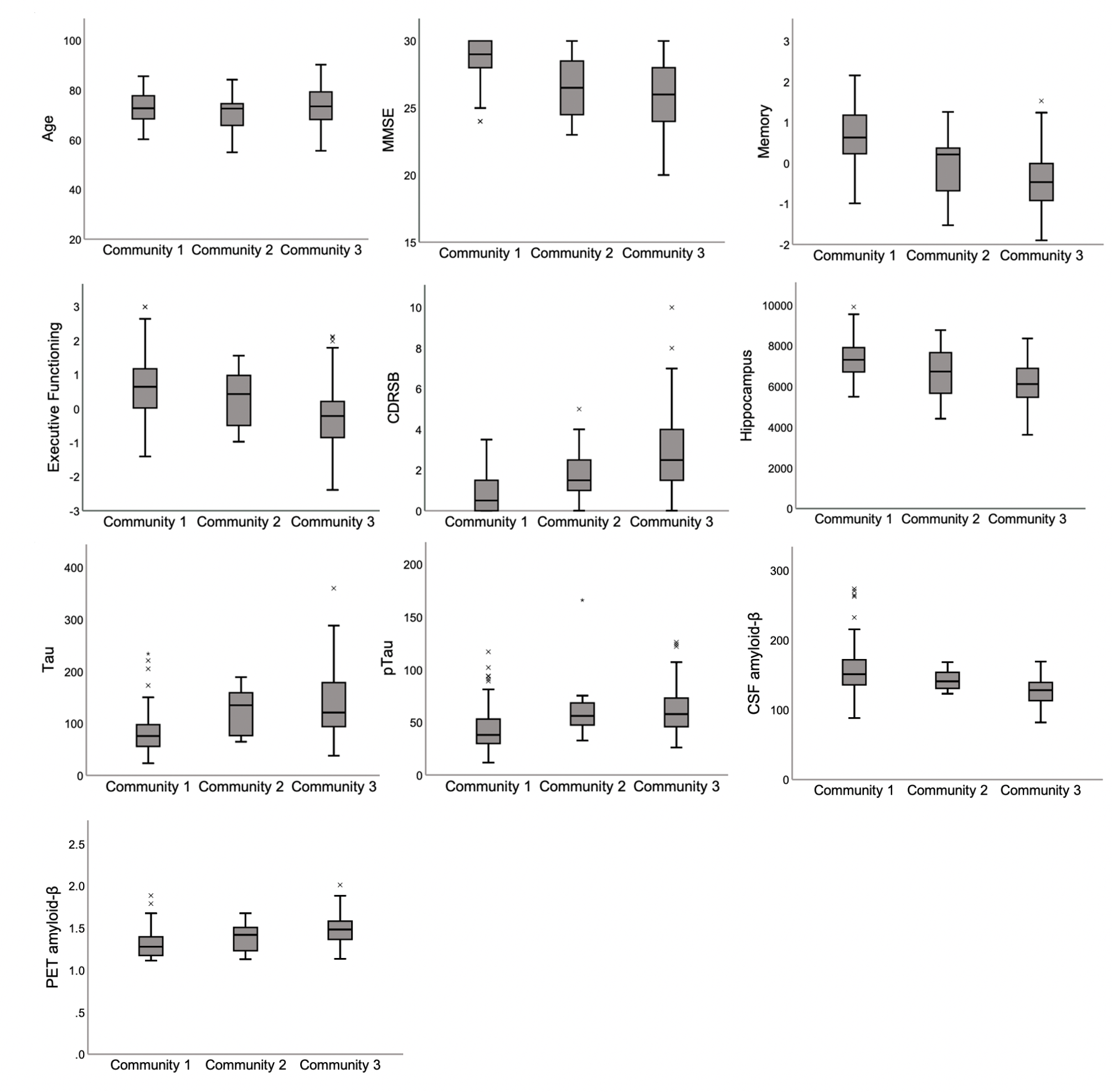
**

**Fig. S10.** Boxplots for amyloid positive subjects in community 1, 2, and 3, for age, MMSE, memory, executive functioning, CDRSB, hippocampal volume, CSF tau, pTau, amyloid-β, and PET amyloid-β.
